# Beyond beneficial or ineffective: three-way decision modeling of image-level affective responses to restorative environment design

**DOI:** 10.3389/fpsyg.2026.1899782

**Published:** 2026-08-19

**Authors:** Qi Li, Chaoyuan Zhang, Qi Li, Ziyu Feng

**Affiliations:** Shanxi University, Taiyuan, China

**Keywords:** affective restoration, biophilic design, cognitive aesthetics, environmental psychology, granular computing, restorative environments, three-way decision, urban green infrastructure

## Abstract

Restorative environments cannot be defined by positive and negative emotion alone. In Attention Restoration Theory, restorative quality depends on being away, fascination, extent/coherence, and compatibility; in Stress Recovery Theory and biophilic design, physiological, safety-related, behavioral, and user-state evidence also matters. Yet, design practice often translates multidimensional evidence into binary beneficial/non-beneficial judgments. Real environments may improve mood while increasing perceived unsafety, reduce arousal while producing boredom, or provide naturalness while remaining socially uncomfortable. To address this problem, this paper proposes the Three-Way Adaptive Restorative Exposure Model (3W-AREM), integrating three-way decision theory, granular computing, and cognitive-aesthetic valuation. The full model defines successful restorative exposure as benefit across affective, attentional, perceptual, physiological, safety, behavioral, and user-state evidence without unacceptable discomfort, overload, unsafety, or avoidance. Cost-sensitive thresholds classify exposure conditions into positive, boundary, and negative regions supporting acceptance, adaptive regulation, and rejection or structural redesign. Granular computing decomposes exposure into medium, environmental type, spatial configuration, sensory composition, parameter intensity, and user-state adaptation. A secondary image-level affective demonstration used an open dataset from a peer-reviewed study on small-scale green infrastructure and affective well-being in urban sites. Because the dataset contains affective image ratings but not perceived restorativeness, ART qualities, attention tasks, physiology, safety ratings, mobility, or embodied exposure, the empirical section operationalizes only one evidence layer of 3W-AREM. Image-level positive and negative affect were used to construct fuzzy membership in an affective-restoration class. Under the main rule, 99 urban-site image conditions were classified into three positive, 87 boundary, and nine negative cases. Green-intervention images showed higher membership than control/placebo images, but most remained in the boundary region. These findings illustrate, rather than validate, that many urban green interventions are mixed affective exposure conditions requiring adaptive diagnosis, optimization, and re-testing.

## 1 Introduction

Built environments increasingly carry responsibility for supporting psychological well-being. Urban densification, work-related stress, digital fatigue, and mental-health challenges have intensified interest in environments that can reduce stress, restore attention, improve affect, and support everyday recovery. In environmental psychology, this concern has been developed through stress recovery theory, attention restoration theory, restorative environment research, and more recent work on biophilic design. Across these traditions, natural and nature-like environments are not understood merely as decorative settings. They are psychologically active conditions that can shape affective, cognitive, and physiological states through perception, bodily regulation, memory, expectation, and environmental appraisal.

Recent evidence supports the importance of this research direction. Systematic reviews and meta-analyses show that nature-based interventions can reduce negative affect, stress, anxiety, depressive symptoms, and physiological arousal, while also enhancing positive affect and relaxation. Research on natural environments, virtual nature, video-based exposure, urban green infrastructure, and indoor biophilic design suggests that restorative experience can be delivered through multiple environmental media and design formats. These findings have encouraged architects, urban designers, healthcare designers, workplace designers, and public-space planners to incorporate vegetation, daylight, water, natural materials, and restorative spatial settings into design practice.

Recent methodological developments also show that restorative-environment research is becoming more computational, multimodal, and visually analytic. Vision-language-model work on urban restorative quality, graph-learning analysis of urban park landscapes, recent EEG studies of virtual coastal or forest landscapes, and design-integrated neuroarchitectural frameworks all point toward a shift from simple group comparison toward pipeline diagrams, spatial evidence maps, network models, uncertainty displays, and psychophysiological dashboards (Ma and Kwan, 2026; Zhang et al., 2026; Shi et al., 2025; Wei et al., 2026). This methodological shift is important for the present study because a three-way model should not only classify environments; it should also display how classification evidence, uncertainty, design granules, and future neurophysiological indicators connect.

**Figure 1:**
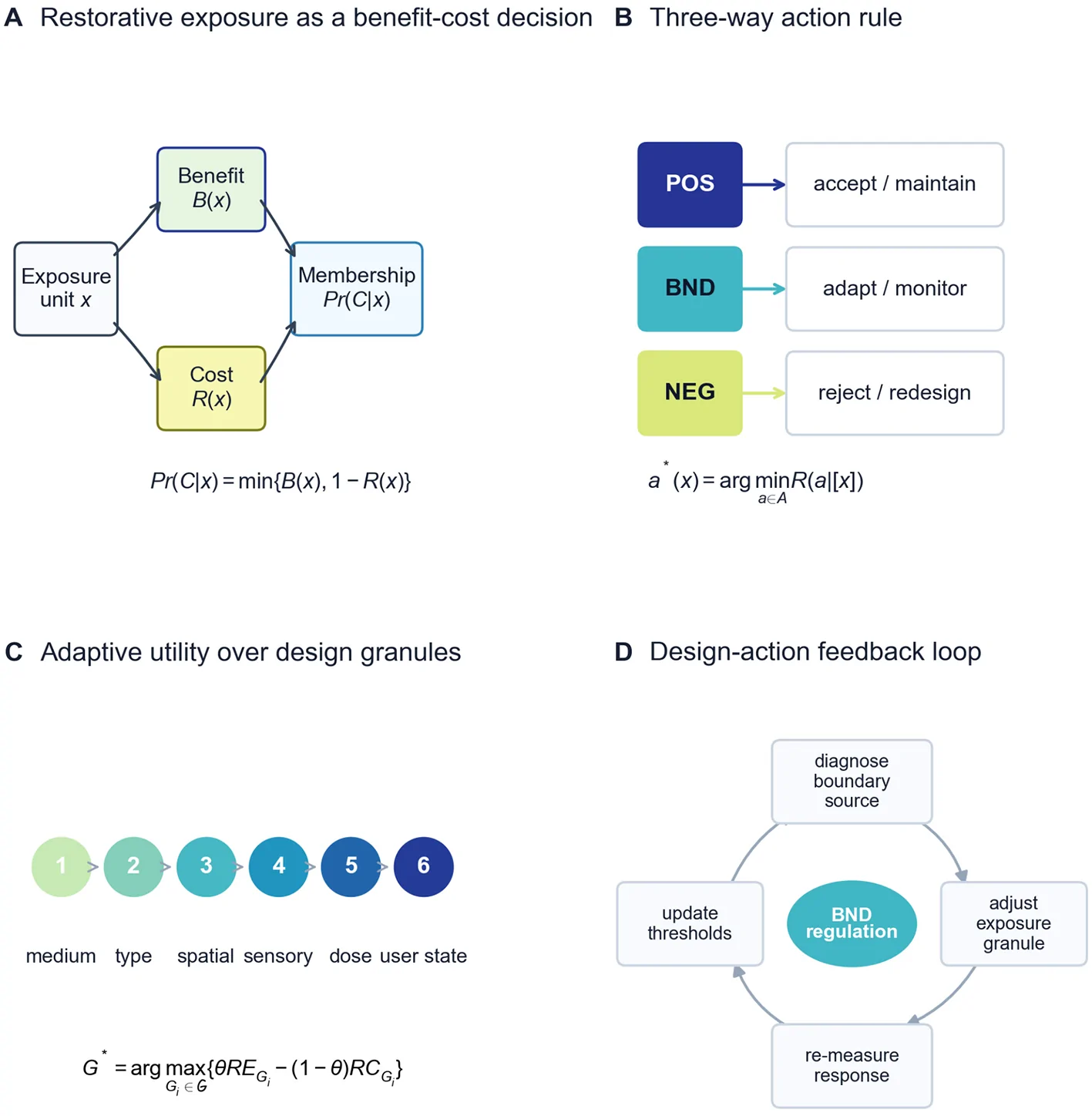
Theoretical architecture of 3W-AREM, including the **(A)** benefit-cost membership structure, **(B)** three-way action rule, **(C)** adaptive design-granule utility, and **(D)** boundary-region feedback loop.

Figure 1 summarizes the overall architecture of the proposed framework before the detailed theoretical and empirical sections are introduced.

However, the accumulated evidence does not support a simple conclusion that more natural exposure is always better or that every biophilic intervention is automatically restorative. Recent meta-analytic and empirical work indicates that restorative effects vary according to exposure duration, environmental type, sensory composition, spatial layout, user characteristics, and measurement method. Attention restoration may be moderated by exposure duration rather than increasing linearly with time. Physiological stress recovery may be detectable in some measures but not in others. Virtual nature may improve affect for some users while producing weak effects, discomfort, or cybersickness for others. These patterns suggest that restorative exposure should be treated as a regulated environmental intervention rather than as a fixed category.

In physical environments, restorative response is also shaped by design trade-offs. Dense vegetation may increase naturalness and fascination but reduce perceived safety if visibility is poor. Water sound may support relaxation in one context but become distracting in another. High visual complexity may invite exploration but also increase cognitive load. Low-stimulation environments may calm anxious users but fail to engage users suffering from attentional fatigue. Urban green spaces with abundant tree cover may still be perceived as unsafe when physical disorder, social disorder, poor maintenance, or low visibility are present. These examples indicate that restorativeness is not a fixed property of green quantity. It is a user-environment response shaped by benefit, cost, and context.

Despite this complexity, restorative environmental design is often translated into practice through a binary logic. Environments are described as restorative or non-restorative, beneficial or ineffective, preferred or non-preferred. Binary interpretation is useful for broad comparison, but it is insufficient for design decision-making. In real design situations, many environments produce mixed responses. A hospital courtyard may reduce stress but feel too exposed. A pocket park may be visually rich but socially unsafe. A VR forest may improve mood but increase cybersickness. A quiet indoor biophilic room may lower arousal but also feel monotonous. Such cases should not be forced into acceptance or rejection. They represent a third state: restorative potential under uncertainty.

This third state is especially important because it identifies environments that are not yet successful but are still design-actionable. If the source of uncertainty can be located, designers may adjust vegetation density, visibility, sound level, exposure duration, spatial enclosure, lighting, maintenance, or user-specific parameters. Mixed restorative responses should therefore be treated not as noise or failure, but as a theoretically meaningful and practically valuable category. This is the key problem addressed in this paper.

Three-way decision theory provides a suitable structure for this problem. Unlike binary classification, three-way decision divides uncertain objects into a positive region, a negative region, and a boundary region. The positive region supports acceptance, the negative region supports rejection, and the boundary region supports deferred decision, adaptive action, or further evaluation. This logic is appropriate for restorative environmental design because the cost of decision errors is asymmetric. Accepting a harmful or uncomfortable exposure pattern as restorative may be more costly than temporarily regulating an uncertain one. Conversely, rejecting a potentially restorative environment too early may waste design resources and overlook opportunities for adaptive improvement.

This paper proposes the Three-Way Adaptive Restorative Exposure Model (3W-AREM), a decision-theoretic framework for classifying and regulating restorative environmental exposure under psychological state uncertainty. The model defines successful restorative exposure as a state in which psychological benefit is achieved without unacceptable discomfort, overload, perceived unsafety, or avoidance. It then uses three-way decision theory to classify exposure patterns into positive, boundary, and negative regions, and uses granular computing to guide adaptive regulation of boundary-region environments. The model is illustrated through a secondary analysis of an open dataset on small-scale green infrastructure and affective well-being in urban sites.

**Figure 2:**
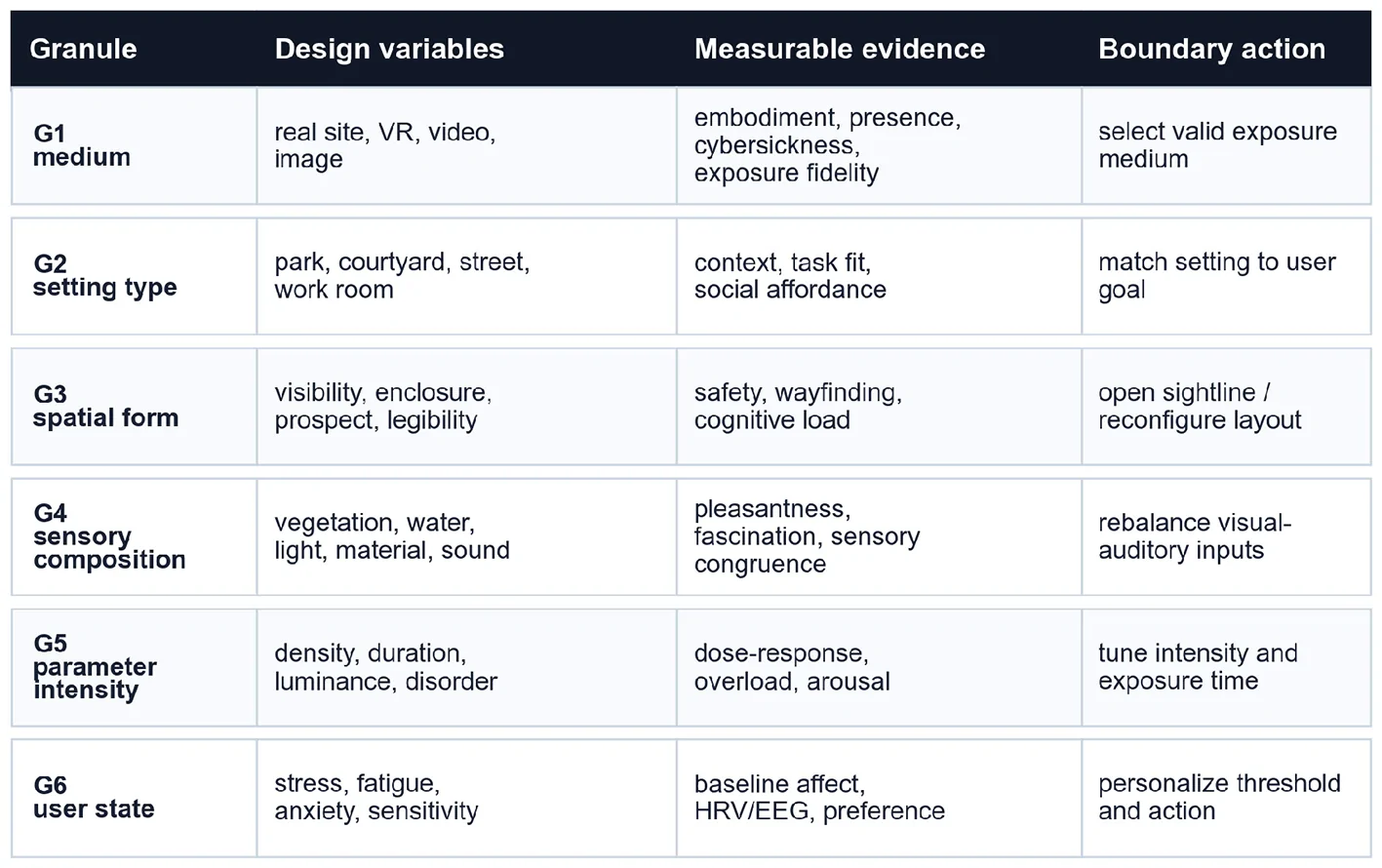
Multi-granularity design-evidence matrix for restorative exposure. The matrix links each design granule to measurable evidence and a boundary-region design action. Interpretation: boundary cases are not simply uncertain; each case points to a design granule that can be adjusted and re-tested.

The paper makes four contributions. First, it reframes restorative environmental design from a binary beneficial/non-beneficial problem into a three-way decision problem. Second, it identifies mixed restorative response as a design-actionable boundary region rather than as methodological noise. Third, it introduces a cost-sensitive model that considers the asymmetric consequences of falsely accepting harmful exposure or falsely rejecting potentially restorative exposure. Fourth, it provides a multi-granularity structure for translating environmental psychology evidence into adjustable design parameters. A secondary neuroaesthetic extension is included as a future research route rather than as an empirical neural contribution of the present paper. Figure 2 previews the granularity logic used throughout the manuscript by linking design granules to evidence types and boundary-region actions.

## 2 Literature review

### 2.1 Restorative environments and biophilic design

Restorative environment research has developed from the broader recognition that environments can influence affective, cognitive, and physiological states. Stress Recovery Theory emphasizes rapid affective and physiological recovery in response to non-threatening natural environments (Ulrich et al., 1991). Attention Restoration Theory emphasizes the recovery of directed attention through environmental qualities such as being away, fascination, extent, and compatibility (Kaplan, 1995; Hartig et al., 1997). Field and attention studies further show that restoration can be evaluated through psychological and cognitive outcomes, not merely through visual preference (Hartig et al., 2003; Berto, 2005). Together, these perspectives have shaped research on parks, forests, gardens, healthcare landscapes, urban green spaces, and indoor environments with natural elements.

Biophilic design extends these ideas into architectural and environmental practice by proposing that natural features, natural analogs, daylight, water, vegetation, material textures, and spatial qualities can support human well-being (Bratman et al., 2019; Sedghikhanshir and Montelli, 2026). The strength of this movement is that it translates environmental psychology into actionable design language. The theoretical literature on biophilic design is not simply decorative or naive; it includes sophisticated accounts of nature contact, spatial experience, sensory variation, place attachment, and human biological needs. The risk addressed in the present paper concerns simplified design implementation, in which nature-based features are treated as generally beneficial ingredients without sufficient attention to intensity, context, user state, and possible adverse responses.

Recent evidence confirms the importance of restorative and nature-based interventions while also revealing their complexity. A systematic overview and second-order meta-analysis of nature-based interventions found beneficial effects for outcomes such as stress, anxiety, depressive symptoms, heart rate, negative affect, positive affect, and relaxation (Robat et al., 2026). Recent critical and scoping reviews further show that nature-based and biophilic interventions differ by interaction mode, setting, user group, intervention property, and evidence channel (Stonebridge et al., 2025; Sedghikhanshir and Montelli, 2026). This level of evidence supports the general claim that nature-based exposure can contribute to psychological and physiological well-being. At the same time, such reviews also highlight heterogeneity in interventions and control conditions, suggesting that nature exposure should not be treated as a single uniform treatment category.

This creates the first gap for the present study. Existing evidence supports restorative environmental exposure, but it does not provide a sufficiently precise decision framework for determining when an exposure pattern should be accepted, rejected, or adaptively modified. The proposed model addresses this gap by treating restorative exposure as a decision problem under uncertainty.

For conceptual clarity, the present manuscript distinguishes four related but non-identical terms (Table 1). This distinction is essential because the present empirical demonstration uses affective ratings only, whereas the full 3W-AREM framework is designed for multidimensional restorative-environment assessment.

**Table 1:** Core concepts used in the proposed 3W-AREM framework.

Concept	Authoritative basis	Use in the present manuscript
Restorative environment	Stress Recovery Theory and Attention Restoration Theory	A multidimensional environment-user relation involving affective, attentional, physiological, perceptual, safety, behavioral, and compatibility evidence. It is not equivalent to positive emotion alone.
Affective restoration	PANAS-like affective change, stress-recovery affect, and affective well-being research	A narrower evidence layer indexed by increased positive affect and reduced negative affect. It is used only in the secondary image-level demonstration.
Restorative potential	Evidence-based design and restorative-environment evaluation	A provisional state in which some indicators support restoration but other indicators are missing, weak, or costly. It motivates boundary-region diagnosis rather than direct acceptance.
Boundary region	Three-way decision theory and probabilistic rough sets	A design-actionable region between acceptance and rejection. It indicates that the environment should be diagnosed, optimized, and re-tested rather than treated as failed.

The same distinction applies to Attention Restoration Theory. In the full model, the four classical ART qualities are not optional background concepts; they are candidate evidence dimensions that should be measured whenever the research question concerns complete restorative recovery (Table 2). Positive affect may accompany these qualities, but it cannot replace them. Therefore, the present open-data demonstration is intentionally labeled an affective demonstration, not a full ART validation.

**Table 2:** Mapping ART restorative qualities to 3W-AREM evidence dimensions and design diagnosis.

ART quality	Recommended evidence	Boundary-region diagnosis in 3W-AREM
Being away	PRS/RPRS items, perceived escape, mental-distance ratings, and exposure context	The environment may feel pleasant but fail to create psychological distance from routine demands. Regulate spatial separation, acoustic shielding, or exposure framing.
Fascination	Soft-fascination ratings, eye-tracking engagement, visual attention, and scene complexity	The environment may be safe but under-engaging, or engaging but overstimulating. Adjust sensory richness, novelty, complexity, and natural detail.
Extent/coherence	Coherence, legibility, mystery, spatial continuity, and navigation confidence	The environment may contain natural elements but lack a coherent restorative scene. Adjust layout, visual continuity, paths, landmarks, and enclosure.
Compatibility	Goal-environment fit, perceived affordance, privacy/social-choice ratings, and behavioral observation	The environment may be visually restorative but mismatched to user needs. Adjust seating, privacy, activity affordances, accessibility, and user-specific exposure dose.

### 2.2 Dose-response and heterogeneity in restorative exposure

One of the most important challenges in restorative environmental design is the problem of dose. In practice, design interventions are often described by the presence or absence of natural elements, such as vegetation, water, daylight, or natural material. However, psychological recovery depends not only on whether such elements exist, but also on their duration, intensity, spatial arrangement, sensory quality, and fit with the user's state.

Recent meta-analytic work on nature exposure and attention restoration provides direct support for this point. Evidence suggests that exposure duration moderates attentional recovery and that the effect is not necessarily linear (Bell et al., 2025). More exposure is not automatically better. An exposure that is too brief may be insufficient, whereas longer exposure may not always produce proportional improvement. This is crucial for design because restorative environments are often experienced in time-limited situations, such as hospital waiting rooms, campus rest spaces, work break areas, or urban pocket parks.

Physiological evidence also shows heterogeneity. A recent meta-analysis of physiological stress responses to natural environments found small-to-moderate stress-reduction effects, but also reported uncertainty concerning exposure type, participant health status, and the relative sensitivity of different physiological measures (Gaekwad et al., 2023). This means that even when natural environments generally support recovery, the strength and detectability of recovery vary across measurement channels and user groups.

The same issue appears in virtual and mediated restorative environments. A systematic review of virtual nature found evidence for effects on mood, stress, and perceived restorativeness, but also noted that optimal exposure type, duration, and individual differences remain underdeveloped (Spano et al., 2023). Recent immersive and indoor studies using virtual, office, hospital-lobby, and window-view paradigms show the growing importance of multimodal psychophysiological evaluation (Yin et al., 2020; Sella et al., 2025; Hasebe and Harada, 2025; Liu and Lee, 2026; Li et al., 2026). Video-based restorative environments offer a feasible alternative to immersive VR, but they also require better specification of exposure conditions and mechanisms. These findings suggest that restorative exposure should be modeled as a regulated intervention rather than a fixed environmental category.

This creates the second gap. Existing studies increasingly recognize dose, duration, medium, and heterogeneity, but there is still a lack of formal decision logic for classifying mixed or uncertain exposure effects. A three-way decision approach can fill this gap by distinguishing positive-region acceptance, negative-region rejection, and boundary-region conditions requiring adaptive regulation.

### 2.3 Design trade-offs: safety, complexity, multisensory interaction, and social affordance

Restorative response is not determined by naturalness alone. Empirical studies in urban parks and green spaces show that natural elements interact with perceived safety, maintenance, visibility, soundscape, social affordance, and spatial configuration. These interactions are central to design because they reveal why an environment can be partly restorative and partly problematic.

Perceived safety is one of the most important constraints. Urban green spaces with vegetation and natural qualities may support mental well-being, but if they are poorly maintained, visually obstructed, socially disordered, or perceived as unsafe, their restorative potential can be reduced. Recent work on urban park qualities shows that perceived safety and naturalness jointly influence perceived restorativeness and mental well-being (Felappi et al., 2024). Other research indicates that physical and social disorder, poor visibility, and perceived crime can reduce perceived safety in green spaces, cautioning against simplistic assumptions that tree cover alone is sufficient.

Spatial and social affordances also matter. A park can feel restorative when users are able to choose how to interact with other people, regulate privacy, and select positions that fit their desired social exposure (Lis et al., 2024). Conversely, the same green space can feel uncomfortable if the user cannot control visibility, distance, or social interaction. This suggests that restorative design should include behavioral choice as a design variable, not only vegetation or visual aesthetics.

Multisensory interaction provides a direct conceptual basis for the evidence-vector model developed later in Equation 1a. Visual, auditory, physiological, and self-report indicators do not always move together. For example, Ha and Kim (2021) study of campus landscape biodiversity showed that visual biodiversity and natural sound can interact, but the interaction pattern depends on the outcome being evaluated. Sakimura et al. (2025) similarly showed that removing visual nature information weakened subjective perceived-restorativeness ratings, while physiological indicators based on HRV and EDA were not degraded in the same way. These findings are important because they show that a single outcome measure can overstate or understate restoration depending on which sensory channel and response system is measured. A visually attractive park may be undermined by traffic noise, excessive water sound, or acoustic incongruence; conversely, a non-visual or weakly visual exposure may still produce physiological regulation. Scene qualities such as coherence, legibility, mystery, and complexity should therefore be interpreted as design-regulable variables rather than fixed aesthetic labels (Stamps, 2004).

This creates the third gap. Environmental design research has identified many variables that affect restoration, but the field still lacks a framework for dealing with mixed trade-offs across outcome channels. A green space may be natural but unsafe, fascinating but cognitively demanding, quiet but boring, physiologically calming but subjectively low in PRS, or immersive but uncomfortable. Such cases require a model that can preserve discrepant evidence across affective, ART, attentional, physiological, safety, behavioral, and user-state indicators rather than forcing them into a binary judgment.

### 2.4 Cognitive aesthetics, sensory valuation, and brain connectivity

The present study also draws on cognitive aesthetics and neuroaesthetics. Environmental restoration is not only a functional response to environmental affordances. It is also a process of sensory valuation, in which perceivers evaluate the environment through sensory input, affective state, learned expectations, bodily condition, and contextual goals. Cognitive-aesthetic models emphasize that aesthetic experience emerges from interactions among perceptual fluency, sensory processing, valuation, physiological state, and behavioral context (Reber et al., 2004; Leder et al., 2004; Nadal and Skov, 2025).

This perspective is useful for restorative environmental design because it shifts the focus from objective environmental features to user-environment valuation. The same environment may be calming for a user seeking solitude, uncomfortable for a user concerned about safety, and boring for a user needing stimulation. Restorativeness should therefore be understood as a context-sensitive valuation outcome rather than a fixed property of natural elements. It is also important to distinguish visual naturalness, aesthetic preference, pleasure, and restoration. A beautiful or highly natural scene may be preferred without necessarily restoring directed attention, reducing stress physiology, or improving behavioral functioning. Conversely, an environment may be moderately preferred but still useful if it reduces overload or supports self-regulation. This distinction is central to the proposed benefit-cost formulation.

Neurophysiological research in architectural and environmental design offers potential measurement tools for this valuation process. EEG, eye-tracking, heart-rate variability, skin conductance, fNIRS, and other measures may help identify responses that are not captured by self-report alone (Fu et al., 2026; Liu and Zhou, 2025; Wei et al., 2026). However, recent reviews caution that neurophysiological design research remains methodologically heterogeneous and should not be treated as a direct replacement for psychological measures. For this reason, the present model treats physiological and neural data as complementary indicators that can help classify uncertain cases, especially when subjective and behavioral measures conflict.

Brain connectivity is particularly relevant for future development of the model. A restorative environment may involve coordination among visual processing networks, attentional control networks, threat and safety appraisal systems, valuation regions such as orbitofrontal and ventromedial prefrontal areas, and memory/meaning networks. Boundary-region environments may be hypothesized to show unstable or competing connectivity patterns: for example, high visual engagement combined with threat-related activation, or valuation signals that are weakened by cognitive load. This paper therefore includes a hypothesis-generating brain-connectivity extension that can be linked to future EEG, fMRI, or source-level connectivity analyses.

### 2.5 Three-way decision theory and granular computing

Three-way decision theory was developed to address uncertain classification and decision-making problems by dividing objects into three regions: positive, boundary, and negative (Yao, 2011, 2016, 2010). The positive region supports acceptance, the negative region supports rejection, and the boundary region supports deferred decision or further action. Compared with binary classification, three-way decision theory is particularly useful when decision errors have asymmetric costs or when available evidence is incomplete, mixed, or uncertain.

Restorative environmental design fits this structure well. Accepting a non-restorative or harmful environment as restorative can produce practical and ethical costs, especially in settings such as hospitals, elder-care facilities, schools, and workplaces. Rejecting a potentially restorative environment too early can also waste resources and overlook design opportunities. A boundary decision allows designers to adapt, monitor, or adjust exposure conditions rather than immediately accepting or rejecting them.

Granular computing complements this decision logic by representing complex problems at multiple levels of resolution (Yao, 2016). In environmental design, this means that a restorative exposure can be analyzed not only as a whole environment, but also through exposure medium, environmental type, spatial configuration, sensory composition, parameter intensity, and user-state adaptation. This multi-level representation is necessary because the source of a mixed response may occur at different scales. A problem may arise from the medium itself, from the spatial layout, from sensory intensity, or from mismatch with the user's current psychological state.

The proposed Three-Way Adaptive Restorative Exposure Model integrates these two ideas. Three-way decision theory identifies whether an exposure pattern should be accepted, rejected, or adaptively regulated. Granular computing identifies which environmental or user-related granules should be adjusted. Together, they provide a formal route from mixed psychological evidence to design action.

Table 3 translates the main restorative-environment constructs into benefit indicators, cost indicators, moderators, and measurement approaches used in the proposed 3W-AREM framework.

**Table 3:** Conceptual translation from restorative-environment evidence to 3W-AREM variables and design operations.

Construct domain	Benefit indicators	Cost or constraint indicators	Moderators and design-operation variables	Measurement approaches
Affective recovery	Positive affect, relaxation, calmness, pleasure, and reduced negative affect	Fear, irritation, tension, avoidance, and boredom when stimulation is too low	Vegetation quality, seating invitation, maintenance, color/material warmth, and exposure duration	PANAS, affect grids, semantic differential ratings, and ecological momentary assessment
Attention and cognition	Directed-attention recovery, fascination, legibility, compatibility, and reduced mental fatigue	Cognitive overload, confusion, excessive complexity, and low wayfinding clarity	Prospect-refuge balance, path clarity, visual complexity, task demand, and user fatigue state	PRS/RPRS scales, attention tasks, eye tracking, and subjective workload
Physiological regulation	Lower heart rate, increased HRV, reduced skin conductance, and lower stress arousal	Hyperarousal, cybersickness, vestibular discomfort, and sensory stress	Soundscape, luminance, temperature, VR field of view, movement speed, and sensory intensity	HR/HRV, EDA, respiration, salivary cortisol, and cybersickness scales
Safety and social comfort	Perceived safety, control, privacy regulation, and comfortable social affordance	Unsafety, low visibility, disorder, crowding, and unwanted social exposure	Lighting, sightlines, maintenance, territorial cues, seating distance, and escape routes	Safety ratings, CPTED audits, behavioral mapping, and visibility/spatial analysis
Aesthetic and meaning valuation	Beauty, coherence, place meaning, nature connectedness, and sensory fluency	Incongruence, alienation, low compatibility, and excessive novelty	Material authenticity, cultural meaning, coherence, mystery, familiarity, and narrative cues	Aesthetic judgment, meaning scales, interviews, and VLM or image-feature analysis

## 3 The three-way adaptive restorative exposure model

### 3.1 Target concept

Let (*U*) denote a universe of restorative exposure units. An exposure unit may be a physical environment, a rendered scene, a VR environment, a video-based exposure, an image condition, or a specific user-environment interaction episode. Let (*C*) denote the target concept of successful restorative exposure.

In this model, an exposure unit belongs to (*C*) when it produces psychological restoration while satisfying basic comfort and safety constraints. Successful restorative exposure is therefore not defined only by positive outcomes. It is defined by the conjunction of benefit and acceptability.

Positive restorative indicators may include increased positive affect, reduced negative affect, reduced perceived stress, improved attentional performance, increased perceived restorativeness, physiological down-regulation, and the four ART qualities of being away, fascination, extent/coherence, and compatibility. Constraint indicators may include perceived safety, low cognitive overload, low sensory discomfort, low avoidance intention, low cybersickness in VR or video exposure, and sufficient environmental legibility.

The target concept is intentionally broader than the empirical instantiation used later in this article. In the full 3W-AREM framework, *B*(*x*) may combine affective, attentional, physiological, behavioral, and self-report evidence, while *R*(*x*) may combine discomfort, unsafety, sensory overload, avoidance, and other constraints. In the secondary data demonstration, however, the available dataset only supports an image-level affective instantiation: positive affect is used as a benefit indicator and low negative affect is used as an affective-cost constraint. This distinction is necessary because affective preference or positive mood should not be equated with complete psychological restoration. The evidence-vector structure and target-concept definition are given in Equations 1a and 1b.

More explicitly, a complete application of 3W-AREM should treat restoration as a vector of evidence rather than a single affective score:


$$\begin{array}{l} \text{E}\left(x\right)=\left\{E_{aff},E_{ART},E_{att},E_{phys},E_{safe},E_{beh},E_{user}\right\} \end{array}$$ (1a)

where *E*_*aff*_ denotes affective evidence, *E*_*ART*_ denotes the ART qualities, *E*_*att*_ denotes attention-task evidence, *E*_*phys*_ denotes physiological regulation, *E*_*safe*_ denotes safety and comfort constraints, *E*_*beh*_ denotes behavioral or usage evidence, and *E*_*user*_ denotes user-state fit. The present dataset only instantiates *E*_*aff*_; therefore, all empirical classifications are described as affective-restoration membership.

Accordingly, the target concept can be expressed conceptually as.


$$\begin{array}{l} C=\left\{x\in U|B\left(x\right)\geq \tau_{B}\;\text{and}\;R\left(x\right)\leq \tau_{R}\right\} \end{array}$$ (1b)

where *B*(*x*) represents the restorative benefit of exposure unit *x*, *R*(*x*) represents the associated risk or discomfort cost, τ_*B*_ is the minimum acceptable benefit threshold, and τ_*R*_ is the maximum acceptable risk threshold. This definition prevents the model from accepting environments that appear restorative on one dimension but fail on another. A densely vegetated park corner may increase fascination but still fail as restorative exposure if it produces low perceived safety.

### 3.2 Three-way classification

Three-way decision theory divides uncertain objects into three regions: Equations 2–4 define the positive, boundary, and negative regionss. In 3W-AREM, these regions are interpreted as environmental design decisions:


$$\begin{array}{l} POS\left(C\right)=\left\{x\in U|Pr\left(C|\left[x\right]\right)\geq \alpha \right\} \end{array}$$ (2)

$$\begin{array}{l} BND\left(C\right)=\left\{x\in U|\beta \leq Pr\left(C|\left[x\right]\right)<\alpha \right\} \end{array}$$ (3)

$$\begin{array}{l} NEG\left(C\right)=\left\{x\in U|Pr\left(C|\left[x\right]\right)<\beta \right\} \end{array}$$ (4)

where [*x*] is the equivalence class of exposure unit *x*, *Pr*(*C*∣[*x*]) is the conditional probability or membership degree that the exposure unit belongs to the target restorative class, and α and β are decision thresholds determined by the loss function.

In classical probabilistic rough-set models, [*x*] denotes an equivalence class induced by available attributes, and *Pr*(*C*∣[*x*]) is estimated as a conditional probability within that class. In the present design framework, the same notation is used more generally because restorative-environment evidence may be probabilistic, fuzzy, or hybrid. When repeated observations are available for environmental classes, *Pr*(*C*∣[*x*]) can be estimated probabilistically. When evidence is built from continuous psychological indicators, it can be instantiated as a fuzzy membership degree μ_*C*_(*x*). The secondary demonstration follows the second route: the image condition is treated as the elementary granule, so [*x*] = *x* for classification, and *Pr*(*C*∣*x*) is used as a three-way-decision-compatible shorthand for μ_*C*_(*x*) rather than as a strict rough-set frequency estimate.

**Figure 3:**
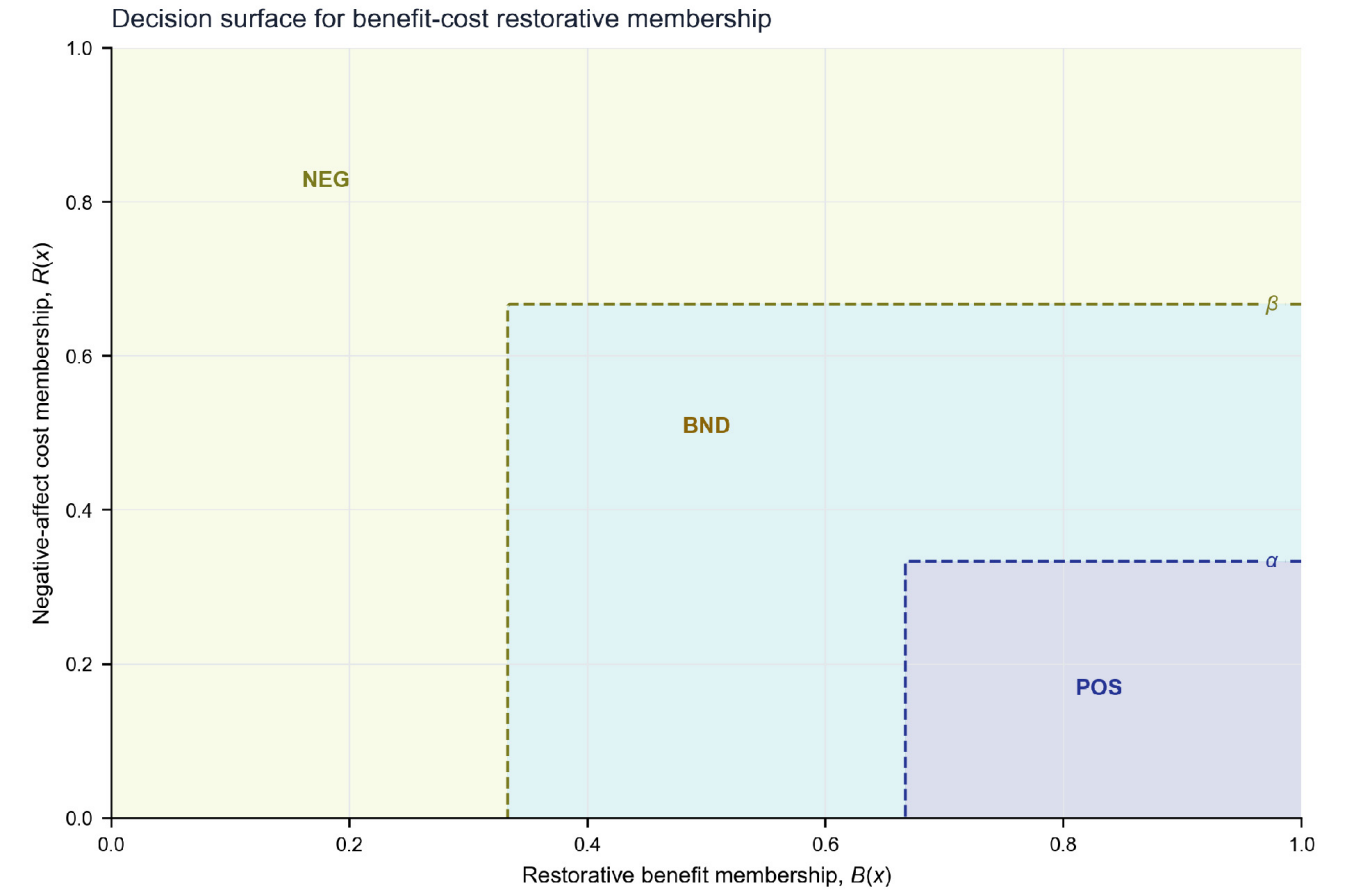
Benefit-cost decision surface for successful restorative exposure. The horizontal axis represents restorative benefit *B*(*x*) and the vertical axis represents affective, cognitive, physiological, or safety-related cost *R*(*x*); cost-sensitive thresholds divide exposure units into positive, boundary, and negative action regions.

The positive region contains exposure units that can be accepted as effective restorative environments. The negative region contains exposure units that should be rejected or structurally redesigned. The boundary region contains exposure units with uncertain or mixed restorative responses. This is the central region of the proposed model. Boundary exposure may produce partial benefit but also partial risk. It may also show inconsistent evidence across measurement channels, such as self-report improvement without physiological recovery, or physiological down-regulation without perceived comfort. In design terms, this region should not be treated as failure. It is the most important region for adaptive regulation. Figure 3 visualizes the resulting benefit-cost decision surface used to classify exposure units before the loss-function thresholds are specified.

### 3.3 Loss function and decision thresholds

The value of three-way decision theory lies in its cost-sensitive treatment of uncertainty. In restorative environmental design, different decision errors have different consequences. Equations 5, 6 define the action and state sets:


$$\begin{array}{l} A=\left\{a_{P},a_{B},a_{N}\right\} \end{array}$$ (5)

where *a*_*P*_ denotes accepting the exposure pattern as restorative, *a*_*B*_ denotes adaptive regulation or deferred decision, and *a*_*N*_ denotes rejecting or structurally redesigning the exposure pattern. The state set is:


$$\begin{array}{l} \Omega =\left\{C,C^{c}\right\} \end{array}$$ (6)

where *C* means that the exposure pattern is successfully restorative and *C*^*c*^ means that it is not. The losses λ_*PP*_, λ_*BP*_, λ_*NP*_, λ_*PN*_, λ_*BN*_, and λ_*NN*_ correspond respectively to the costs of accepting, regulating, or rejecting exposure patterns under restorative and non-restorative states.

In evidence-based environmental design, the most serious error is accepting an exposure pattern that is actually non-restorative or harmful. A design intervention may be promoted as restorative while increasing unsafety, overload, or discomfort in vulnerable users. Therefore, λ_*PN*_ should generally be set relatively high. Equations 7, 8 state the loss-ordering assumptions:


$$\begin{array}{l} 0\leq \lambda_{PP}\leq \lambda_{BP}\leq \lambda_{NP} \end{array}$$ (7)

$$\begin{array}{l} 0\leq \lambda_{NN}\leq \lambda_{BN}\leq \lambda_{PN} \end{array}$$ (8)

Equations 9–12 define expected losses and the minimum-loss decision rule:


$$\begin{array}{l} R\left(a_{P}|\left[x\right]\right)=\lambda_{PP}Pr\left(C|\left[x\right]\right)+\lambda_{PN}Pr\left(C^{c}|\left[x\right]\right) \end{array}$$ (9)

$$\begin{array}{l} R\left(a_{B}|\left[x\right]\right)=\lambda_{BP}Pr\left(C|\left[x\right]\right)+\lambda_{BN}Pr\left(C^{c}|\left[x\right]\right) \end{array}$$ (10)

$$\begin{array}{l} R\left(a_{N}|\left[x\right]\right)=\lambda_{NP}Pr\left(C|\left[x\right]\right)+\lambda_{NN}Pr\left(C^{c}|\left[x\right]\right) \end{array}$$ (11)

The decision rule selects the action with the minimum expected loss:


$$\begin{array}{l} a^{*}\left(x\right)=\arg\underset{a\in A}{\min}R\left(a|\left[x\right]\right) \end{array}$$ (12)

Under standard assumptions, Equations 13, 14 give the resulting decision thresholds:


$$\begin{array}{l} \alpha =\frac{\lambda_{PN}-\lambda_{BN}}{\left(\lambda_{PN}-\lambda_{BN}\right)+\left(\lambda_{BP}-\lambda_{PP}\right)} \end{array}$$ (13)

$$\begin{array}{l} \beta =\frac{\lambda_{BN}-\lambda_{NN}}{\left(\lambda_{BN}-\lambda_{NN}\right)+\left(\lambda_{NP}-\lambda_{BP}\right)} \end{array}$$ (14)

Thus, if *Pr*(*C*∣[*x*])≥α, the exposure pattern is accepted; if β ≤ *Pr*(*C*∣[*x*]) < α, the exposure pattern is adaptively regulated; and if *Pr*(*C*∣[*x*]) < β, the exposure pattern is rejected or structurally redesigned.

In environmental-psychological terms, α is the evidentiary standard for saying that an exposure condition is sufficiently beneficial and acceptable to be adopted as a restorative design pattern. A high α is appropriate when false acceptance is ethically costly, for example in hospitals, elder-care settings, mental-health settings, or schools. By contrast, β is the lower evidentiary boundary below which the condition is too weak or too costly to justify minor adjustment. The boundary region between β and α is therefore not a failed model output. It is the formal location of design uncertainty: the exposure may have partial value, but the evidence is not strong enough for acceptance and not weak enough for rejection. The numerical loss values used in the secondary demonstration are illustrative and theory-guided rather than expert-calibrated; future work should estimate them through expert elicitation, stakeholder Delphi panels, risk analysis, Bayesian decision modeling, or context-specific sensitivity analysis.

Table 4 clarifies the design meaning of loss calibration. The exact values are not universal constants. They represent a transparent way to encode the relative seriousness of false acceptance, false rejection, and delayed regulation in a specific design context. This is why threshold sensitivity is not a secondary technical detail but a necessary part of responsible model use.

**Table 4:** Design interpretation of loss calibration in different restorative-environment contexts.

Context	Cost of false acceptance	Cost of false rejection	Threshold implication
Hospital courtyard or lobby	High: discomfort, perceived unsafety, privacy loss, or stress in vulnerable users	Moderate: a potentially useful design may be delayed	Use a relatively high α and a broad boundary region; require re-testing before acceptance.
Urban pocket park	Moderate to high: unsafe or disordered spaces may be mislabeled restorative	Moderate: low-cost improvements may be missed	Use context-specific α and β calibrated by safety, maintenance, visibility, and user group.
Indoor restorative space	Moderate: overstimulation, boredom, poor compatibility, or reduced productivity	Low to moderate: features can often be adjusted iteratively	Allow adaptive boundary tuning using exposure duration, sound level, lighting, and user-state fit.
VR or mediated nature	High for sensitive users: cybersickness, sensory overload, motion discomfort	Moderate: useful mediated exposure may be underused	Increase cost assigned to discomfort indicators and personalize dose thresholds.

### 3.4 Restorative exposure granules

The boundary region becomes useful only if the designer can determine what should be adjusted. For this reason, 3W-AREM integrates granular computing. In granular computing, complex objects are represented at multiple resolutions so that analysis can shift between coarse categories and finer actionable attributes. The six granularity levels used here were not intended as a universal ontology of all possible design variables. They are an operational taxonomy synthesized from restorative-environment research, biophilic and evidence-based design practice, exposure-medium studies, dose-response research, multisensory design, safety/legibility research, and user-state adaptation. They were selected because each level corresponds to a decision point that designers can actually regulate: the medium through which exposure is delivered, the type of setting, spatial organization, sensory composition, parameter intensity, and user-state fit. Instead of treating an environment as a single object, the model decomposes restorative exposure into the following granularity levels:

- GL1: exposure medium, including real environment, VR environment, video exposure, image exposure, or mixed-reality exposure.
- GL2: environmental type, including urban pocket park, campus rest space, hospital courtyard, workplace decompression room, residential green space, or indoor biophilic room.
- GL3: spatial configuration, including openness, enclosure, prospect-refuge condition, path clarity, seating position, visibility, and ability to choose social interaction.
- GL4: sensory composition, including vegetation, water, daylight, shadow, soundscape, material texture, air movement, and visual access to sky.
- GL5: parameter intensity, including vegetation density, visual complexity, luminance, acoustic level, water sound intensity, exposure duration, maintenance condition, and degree of physical or social disorder.
- GL6: user-state adaptation, including baseline stress, mental fatigue, anxiety, age, nature connectedness, sensory sensitivity, prior experience, and health condition.

These categories are therefore illustrative and extensible rather than exhaustive. A field study might add management granules such as maintenance regime, social programming, or seasonal change; a VR study might split GL1 into display fidelity, navigation mode, and interaction device; a healthcare study might add clinical vulnerability or privacy requirements. The important point is not that GL1–GL6 are the only possible granules, but that every boundary-region classification should be linked to an explicit and adjustable design granule.

The granularity levels are also mutually dependent rather than statistically independent. GL1 constrains the evidence that can be collected: an image exposure can support visual-affective ratings but cannot directly measure embodied movement or soundscape. GL2 constrains the acceptable thresholds and relevant risks: a hospital lobby requires different safety and privacy tolerances than a recreational pocket park. GL3 spatial configuration shapes GL4 sensory composition, because visibility, enclosure, and path structure determine how vegetation, water, daylight, and sound are perceived. GL5 parameter intensity tunes the dose of variables introduced at GL3 and GL4, while GL6 user-state adaptation moderates every other level. Table 5 therefore treats the six levels as an ordered diagnostic hierarchy rather than as independent predictors.

**Table 5:** Operational granularity table for translating 3W-AREM classification into design variables and adaptive actions.

Granularity	Main variables	Data type	Possible threshold logic	Boundary-region adaptive action
GL1 Medium	Real site, VR, video, image, and mixed reality	Categorical exposure condition	Medium-specific comfort and ecological-validity rules	Change medium, reduce immersion, add field validation, or match medium to user tolerance
GL2 Environment type	Hospital courtyard, pocket park, campus rest space, and indoor biophilic room	Categorical or typological class	Context-specific α, β and safety tolerance	Select a more appropriate design typology or compare alternatives within the same typology
GL3 Spatial configuration	Visibility, enclosure, prospect-refuge balance, path clarity, and seating control	Spatial metrics, audits, ratings	Minimum visibility, maximum enclosure risk, legibility score	Open blocked sightlines, clarify paths, reposition seating, and increase controllable refuge
GL4 Sensory composition	Vegetation, water, soundscape, daylight, materials, and sky view	Image features, acoustic data, sensor data, ratings	Minimum natural affordance with upper limits for overload	Adjust planting mix, sound level, luminance, shade, material texture, or multimodal congruence
GL5 Parameter intensity	Vegetation density, complexity, exposure duration, noise level, and maintenance/disorder	Continuous design or environmental measures	Dose-response and tolerance thresholds	Tune intensity, duration, maintenance, and complexity until benefit rises without cost increase
GL6 User-state adaptation	Baseline stress, fatigue, anxiety, sensory sensitivity, age, and nature connectedness	Individual-level psychometric, behavioral, or physiological data	Personalized *RE*_*user*_ and *RC*_*user*_ constraints	Personalize exposure dose, route, seating position, VR intensity, or sensory channel

This granularity structure allows the model to identify whether a boundary-region exposure should be adjusted globally or locally. If a courtyard produces low restoration because of excessive enclosure and poor visibility, the problem belongs mainly to spatial configuration. If the same courtyard produces high fascination but sensory overload, the problem may belong to sensory composition or parameter intensity.

For exposure units in the boundary region, the design task is to search for an adjusted exposure granule *G*_*i*_ that increases restorative benefit while reducing discomfort or risk. Let *RE*_*G*_*i*__ denote the restorative effect of granule *G*_*i*_, and let *RC*_*G*_*i*__ denote the restorative cost, including discomfort, insecurity, cognitive load, or sensory overload. Equations 15–18 define adaptive utility, optimal granule selection, and user-level benefit and cost constraints:


$$\begin{array}{l} U\left(G_{i}\right)=\theta RE_{G_{i}}-\left(1-\theta \right)RC_{G_{i}} \end{array}$$ (15)

where θ∈[0, 1] represents the relative emphasis on restorative gain versus risk reduction. The design optimization can then be expressed as:


$$\begin{array}{l} G^{*}=\arg\underset{G_{i}\in G}{\max}U\left(G_{i}\right) \end{array}$$ (16)

subject to:


$$\begin{array}{l} RE_{G_{i}}\geq RE_{user} \end{array}$$ (17)

$$\begin{array}{l} RC_{G_{i}}\leq RC_{user} \end{array}$$ (18)

where *RE*_*user*_ is the user's minimum acceptable restorative gain and *RC*_*user*_ is the user's maximum acceptable risk or discomfort. Unlike a narrative-selection model, the purpose here is not to choose the most meaningful statement. The purpose is to adjust the exposure prescription.

In empirical applications, *RE*_*G*_*i*__ can be operationalized as the expected gain produced by changing granule *G*_*i*_, such as an increase in positive affect, perceived restorativeness, attention-task performance, HRV, or relaxation. *RC*_*G*_*i*__ can be measured as the expected cost introduced by the same granule, such as increased negative affect, unsafety, cognitive load, sensory discomfort, cybersickness, or avoidance intention. *RE*_*user*_ and *RC*_*user*_ define user- or setting-specific acceptability constraints. For example, a hospital patient with high anxiety may require a lower maximum safety-related cost than a recreational park user, while a fatigued student may require a higher minimum attentional gain before a campus rest space is accepted.

### 3.5 Operating procedure: evaluation, diagnosis, optimization, and re-testing

The proposed model is intended as an operating procedure rather than a stand-alone classifier. A complete 3W-AREM application should proceed through four linked phases: evaluation, diagnosis, optimization, and re-testing (Table 6). This procedural framing is important because the boundary region is useful only if it leads to a targeted design action.

**Table 6:** Complete operating procedure of 3W-AREM.

Phase	Main operation	Required output
Evaluation	Measure evidence vector **E**(*x*) across affective, ART, attentional, physiological, safety, behavioral, and user-state indicators.	Normalized benefit and cost indicators, with missing evidence channels explicitly marked rather than silently ignored.
Three-way classification	Compute *Pr*(*C*∣[*x*]) or μ_*C*_(*x*) and apply context-specific α and β.	Assignment to positive, boundary, or negative region with transparent threshold settings.
Boundary diagnosis	Locate the binding limitation: benefit-limited, cost-limited, mixed high-benefit/high-cost, or evidence-insufficient.	Diagnostic statement linking the decision region to one or more design granules GL1–GL6.
Optimization	Select the smallest feasible design adjustment that improves the binding limitation without creating new cost.	Scenario-specific intervention plan, such as visibility improvement, sensory-intensity reduction, compatibility enhancement, or dose personalization.
Re-testing	Re-measure the adjusted exposure using the same evidence vector and thresholds.	Acceptance only if the adjusted exposure crosses α without unacceptable cost; otherwise repeat diagnosis or reject/redesign.

The matching between decision regions and granularity dimensions is therefore action-dependent. Positive-region exposures are candidates for acceptance and implementation, but they should still be monitored in higher-risk settings. Negative-region exposures usually require structural redesign at GL2 or GL3 rather than minor parameter adjustment. Boundary-region exposures require the most detailed granule matching because they may be benefit-limited, cost-limited, or evidence-insufficient (Table 7).

**Table 7:** Matching three decision regions with design granules and design action.

Decision region	Diagnostic meaning	Typical granule focus	Design action
Positive region	Benefit is sufficient and cost is acceptable.	GL2–GL6 monitoring depending on context.	Accept as a provisional restorative pattern; monitor safety, use, and user-state fit.
Boundary: benefit-limited	Costs are acceptable but restorative benefit is insufficient.	GL4 sensory composition, GL5 parameter intensity, and GL6 compatibility.	Increase soft fascination, coherence, affordance quality, or user-goal fit; then re-test.
Boundary: cost-limited	Benefit exists but discomfort, unsafety, overload, or avoidance is too high.	GL3 spatial configuration, GL4 sensory composition, and GL5 intensity.	Reduce visual obstruction, noise, complexity, exposure duration, cybersickness, or social discomfort.
Boundary: evidence-insufficient	Available indicators are too narrow or inconsistent.	Measurement granules across *E*_*aff*_, *E*_*ART*_, *E*_*att*_, *E*_*phys*_, and *E*_*safe*_.	Collect PRS/RPRS, attention-task, physiological, safety, or behavioral data before acceptance.
Negative region	Benefit is weak and/or cost is high.	GL2 environmental type and GL3 spatial structure.	Reject as restorative reference or conduct structural redesign before re-evaluation.

### 3.6 Cognitive-aesthetic valuation mapping

From a cognitive-aesthetic perspective, restorative exposure can be modeled as a sensory valuation process. Sensory fluency, beauty, coherence, fascination, and place meaning may contribute to *B*(*x*); threat appraisal, sensory overload, spatial uncertainty, cybersickness, and unwanted social exposure may contribute to *R*(*x*); and user goals, prior experience, health status, and environmental context may shape the weights and thresholds used in classification. This mapping allows aesthetic valuation to enter the model without reducing restoration to beauty, preference, or visual naturalness alone.

This positioning also clarifies the role of neuroaesthetics in the present paper. No EEG, fMRI, fNIRS, eye-tracking, or physiological connectivity data are analyzed in the secondary demonstration. Neural and brain-connectivity measures are therefore not treated as evidence for the current empirical classifications. Instead, they are reserved as an optional future-evidence module that could be added after independent neural or physiological data have been collected, pre-registered, and validated. The formal version of this future module is presented after the limitations section, where the boundary between present findings and future research is made explicit.

## 4 Secondary data demonstration

### 4.1 Purpose and data source

The purpose of this section is to demonstrate how 3W-AREM can be applied to real affective-response data. Rather than using hypothetical environmental exposure units, the demonstration uses an open dataset associated with a peer-reviewed study on small-scale green infrastructure and affective well-being in urban sites. The demonstration is deliberately described as image-level affective classification. It does not claim to validate complete restorative recovery, because the available open data do not include attention restoration tasks, physiological stress recovery, perceived safety, environmental sound, embodied movement, or repeated exposure duration.

The data were obtained from the open dataset by Navarrete-Hernandez and Laffan (2022), “The impact of small-scale green infrastructure on the affective wellbeing associated with urban sites,” deposited on Zenodo. The dataset is associated with a peer-reviewed Scientific Reports article of the same topic (Navarrete-Hernandez and Laffan, 2023). The downloaded data file contained 62,739 affective-response records, 3,567 user IDs, and 99 unique urban-site image conditions. The study used a PANAS-based affective assessment, asking participants how strongly they felt specific positive or negative affective states in relation to public-space images.

Data cleaning followed a conservative reproducibility rule. The analysis retained only records with valid image or site identifiers, treatment labels, affective item labels, user identifiers when available, and affect ratings within the documented 1–10 response range. Affective item strings and treatment labels were standardized before aggregation. No missing affect ratings were imputed, because the goal was not to reconstruct individual PANAS profiles but to create transparent image-level summaries from observed responses. The resulting classification dataset contained 99 image conditions, which are the design granules analyzed in the three-way demonstration.

The demonstration is not intended to re-estimate the causal effects reported in the original study. Instead, it uses the dataset to illustrate how urban-site images can be reclassified into three formal decision regions: positive, boundary, and negative. These regions are interpreted, respectively, as affectively restorative, mixed or uncertain, and affectively adverse for design action. Safety is treated as a theoretical constraint in the full model, but it is not empirically measured in this dataset and is therefore not used as evidence in the present classification.

### 4.2 Analytical unit and affective indicators

Inspection of the dataset showed that participants generally responded to one PANAS-like affective adjective rather than completing the full 20-item PANAS profile for every image. Therefore, the present demonstration uses the image condition as the analytical unit. For each image condition (*x*), responses were aggregated across participants and affective items to estimate mean positive affect, mean negative affect, and fuzzy membership in a successful affective-restoration class.

Positive affect items included optimistic, active, alert, attentive, determined, enthusiastic, strong, inspired, interested, and proud. Negative affect items included scared, ashamed, guilty, angry, hostile, restless, irritable, upset, nervous, and fearful. For each image condition, the mean positive affect score (*PA*(*x*)) and mean negative affect score (*NA*(*x*)) were computed from the available valid item-level responses:


$$\begin{array}{l} PA\left(x\right)=\frac{1}{n_{PA}\left(x\right)}\underset{i\in I_{PA}\left(x\right)}{\sum }y_{i},\;NA\left(x\right)=\frac{1}{n_{NA}\left(x\right)}\underset{i\in I_{NA}\left(x\right)}{\sum }y_{i} \end{array}$$

where *I*_*PA*_(*x*) and *I*_*NA*_(*x*) are the sets of valid positive- and negative-affect responses for image condition *x*, *y*_*i*_ is the 1–10 affect rating, and *n*_*PA*_(*x*) and *n*_*NA*_(*x*) are the corresponding response counts. These counts varied across image conditions because the open dataset was collected through item-level image ratings rather than complete 20-item PANAS profiles for every image. In the processed image-level dataset, *n*_*PA*_(*x*) ranged from 203 to 2313 responses per image (median = 235; mean = 421.9), and *n*_*NA*_(*x*) ranged from 81 to 1084 responses per image (median = 111; mean = 196.3). The total number of retained positive- and negative-affect item responses used in the image-level summaries was 61,204. Equations 19–22 define PA/NA normalization, low-negative-affect membership, and the final affective-restoration membership function.

Because the response scale ranged from 1 to 10, the scores were normalized to the interval ([0,1]):


$$\begin{array}{l} B\left(x\right)=\frac{PA\left(x\right)-1}{9} \end{array}$$ (19)

$$\begin{array}{l} R\left(x\right)=\frac{NA\left(x\right)-1}{9} \end{array}$$ (20)

where (*B*(*x*)) is affective restorative benefit and (R(x)) is affective cost. Low negative affect membership was defined as:


$$\begin{array}{l} LowNA\left(x\right)=1-R\left(x\right) \end{array}$$ (21)

Successful affective restoration requires both positive affective benefit and low negative affective cost. Therefore, the fuzzy membership of image condition (*x*) in the target class (*C*) was defined as:


$$\begin{array}{l} Pr\left(C|x\right)=\min\left(B\left(x\right),LowNA\left(x\right)\right) \end{array}$$ (22)

The fuzzy-min operator is conservative and interpretable. It prevents an image from being classified as successfully restorative if it has high positive affect but also high negative affect, or if it has low negative affect but insufficient positive affective benefit.

In this empirical instantiation, *Pr*(*C*∣*x*) should be read as μ_*C*_(*x*), a fuzzy membership score used within a three-way decision rule. It is not a direct estimate that a randomly sampled person will be restored by the image. This distinction is important because the dataset supports image-level aggregation but not individual-level probability modeling.

Table 8 summarizes the reproducible analytical workflow from data cleaning to threshold classification and robustness checks.

**Table 8:** Reproducible analytical workflow for the secondary image-level affective demonstration.

Step	Operation
1	Import the Zenodo open dataset and retain records with valid image/site identifiers, treatment labels, affective item labels, and 1–10 affect ratings.
2	Map PANAS-like items into positive-affect items (optimistic, active, alert, attentive, determined, enthusiastic, strong, inspired, interested, and proud) and negative-affect items (scared, ashamed, guilty, angry, hostile, restless, irritable, upset, nervous, and fearful).
3	Aggregate responses to the image-condition level and retain response counts *n*_*PA*_(*x*) and *n*_*NA*_(*x*) for each image because the number of valid positive- and negative-affect ratings varied across image conditions.
4	Normalize mean positive affect and mean negative affect to [0, 1], compute *B*(*x*), *R*(*x*), *LowNA*(*x*), and μ_*C*_(*x*) = min(*B*(*x*), *LowNA*(*x*)).
5	Apply the loss-derived thresholds α and β to classify each image into *POS*(*C*), *BND*(*C*), or *NEG*(*C*).
6	Compare treatment groups and site-image categories using descriptive statistics, robust group tests, bootstrap confidence intervals, permutation tests, and three-way region distributions.
7	Evaluate threshold sensitivity and membership-function alternatives to determine whether conclusions depend on a single arbitrary specification.

Alternative membership functions were examined as a specification check. A geometric mean or weighted mean allows high positive affect to partially compensate for lower low-negative-affect membership, while a product rule penalizes low scores more strongly. Under the main thresholds, these alternatives produced different region counts: geometric mean, 18 positive, 81 boundary, and 0 negative cases; equal weighted mean, 22 positive, 77 boundary, and 0 negative cases; benefit-weighted mean with weights 0.60/0.40, 16 positive, 83 boundary, and 0 negative cases; and product rule, 0 positive, 47 boundary, and 52 negative cases. The min rule was retained because the theoretical target concept is conjunctive: an exposure condition should not be accepted as affectively restorative unless both affective benefit and low affective cost are sufficient. The alternatives show that the central finding of substantial boundary-region membership is robust, although the exact number of positive and negative cases depends on how compensatory the membership rule is allowed to be.

### 4.3 Generalizing membership construction beyond PANAS

The PANAS demonstration is only one instantiation of 3W-AREM. In other datasets, the same logic can be applied by first separating benefit indicators from cost or constraint indicators and then normalizing them to comparable membership scales. Let *s*_*j*_(*x*) denote normalized benefit indicators, such as PRS/RPRS, fascination, compatibility, attention-task improvement, HRV increase, or positive affect. Let *c*_*k*_(*x*) denote normalized cost indicators, such as perceived unsafety, cognitive overload, excessive arousal, EDA increase, cybersickness, discomfort, avoidance intention, or sensory incongruence. A general implementation can be written as:


$$\begin{array}{lll} B\left(x\right) & = & A_{B}\left\{s_{1}\left(x\right),s_{2}\left(x\right),\ldots ,s_{m}\left(x\right)\right\},\\ R\left(x\right) & = & A_{R}\left\{c_{1}\left(x\right),c_{2}\left(x\right),\ldots ,c_{q}\left(x\right)\right\} \end{array}$$

$$\begin{array}{l} \mu_{C}\left(x\right)=\min\left\{B\left(x\right),1-R\left(x\right)\right\} \end{array}$$

where *A*_*B*_ and *A*_*R*_ may be a minimum rule, weighted mean, geometric mean, or pre-registered latent score depending on the research question. A conservative restoration study should use a non-compensatory or weakly compensatory rule when false acceptance is costly, because high PRS or high positive affect should not fully compensate for strong unsafety, overload, or physiological stress.

Table 9 gives practical guidance for constructing membership functions when the available evidence is not PANAS. The key principle is that benefit-only measures should not be treated as complete evidence of successful restoration unless a cost or constraint channel is also measured. For example, PRS primarily measures perceived restorative qualities and therefore belongs mainly to *B*(*x*). If a PRS dataset lacks safety, discomfort, overload, or physiological constraint indicators, 3W-AREM should classify the result as restorative potential or evidence-insufficient boundary membership rather than as full acceptance. A cost component can be added through perceived safety, comfort, avoidance intention, workload, EDA/HRV stress markers, cybersickness, or environmental-risk audits.

**Table 9:** Practical guidance for constructing 3W-AREM membership functions beyond PANAS.

Dataset type	Benefit component *B*(*x*)	Cost component *R*(*x*)	Recommended membership logic	Interpretation if cost data are missing
PRS/RPRS or ART scales	Being away, fascination, coherence/extent, compatibility, and perceived restoration	Unsafety, discomfort, incompatibility, overload, avoidance, low privacy, and poor legibility	Combine ART dimensions into *B*(*x*); combine safety/comfort constraints into *R*(*x*); use min{*B*, 1−*R*} for acceptance	Treat as restorative potential or evidence-insufficient boundary, not complete acceptance
Attention-task data	Post-exposure improvement in directed-attention task, lower fatigue, and lower workload	Cognitive overload, confusion, excessive task effort, poor wayfinding, and performance instability	Normalize change scores relative to baseline/control; pair attentional gain with workload or comfort cost	Require complementary subjective or safety evidence before acceptance
Physiological data	HRV increase, heart-rate reduction, lower EDA, lower cortisol, and respiration regulation	Hyperarousal, EDA increase, discomfort, cybersickness, and thermal/acoustic stress	Directionally code physiological recovery as benefit and dysregulation as cost; pre-register baseline and responder rules	Interpret as physiological restoration only, not complete psychological restoration
Multisensory or VR data	PRS, affect, presence, fascination, attention, and physiological regulation	Cybersickness, sensory conflict, overload, motion discomfort, noise annoyance, and low control	Use channel-specific benefit and cost scores; require medium-specific constraints at GL1 and GL5	Place in boundary region until medium-specific adverse effects are measured
Field or design-audit data	Dwell time, return intention, use diversity, perceived restoration, safety, and comfort	Avoidance, low visibility, disorder, crowding, heat/noise stress, and accessibility barriers	Combine behavioral and environmental indicators with self-report; use context-calibrated thresholds	Use diagnostic boundary category if user-state or safety data are incomplete

### 4.4 Decision thresholds

For the secondary demonstration, Equations 23–26 specify the illustrative loss parameters and resulting thresholds:


$$\begin{array}{l} \lambda_{PP}=0,\;\lambda_{BP}=4,\;\lambda_{NP}=8 \end{array}$$ (23)

$$\begin{array}{l} \lambda_{NN}=0,\;\lambda_{BN}=2,\;\lambda_{PN}=10 \end{array}$$ (24)

This setting assumes that falsely accepting an affectively adverse environment as restorative is the most costly error. Falsely rejecting a potentially restorative environment also has cost, but it is less severe than promoting an environment that may increase negative affect. Adaptive regulation has an intermediate cost because it requires further design effort or additional evaluation. The thresholds are:


$$\begin{array}{l} \alpha =\frac{10-2}{\left(10-2\right)+\left(4-0\right)}=0.667 \end{array}$$ (25)

$$\begin{array}{l} \beta =\frac{2-0}{\left(2-0\right)+\left(8-4\right)}=0.333 \end{array}$$ (26)

Thus, if *Pr*(*C*∣*x*)≥0.667, the image condition belongs to *POS*(*C*); if 0.333 ≤ *Pr*(*C*∣*x*) < 0.667, it belongs to *BND*(*C*); and if *Pr*(*C*∣*x*) < 0.333, it belongs to *NEG*(*C*).

In design language, α = 0.667 means that an image condition must reach a relatively high affective-membership score before it is accepted as sufficiently restorative. β = 0.333 means that very weak affective membership triggers rejection or structural redesign. Scores between these thresholds are not ambiguous failures; they are conditions where design benefit is plausible but not yet strong enough for acceptance.

**Figure 4:**
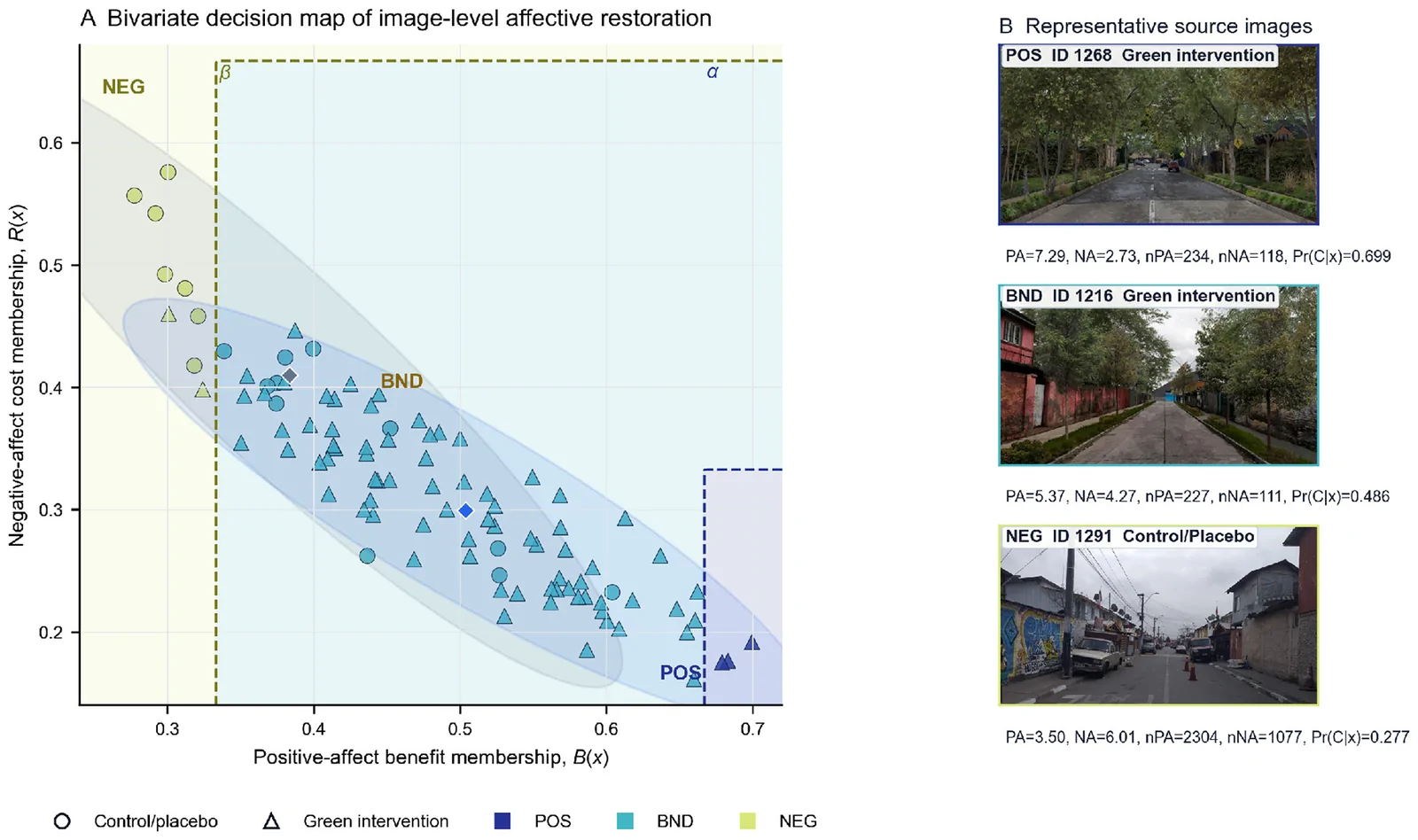
Bivariate decision map of image-level affective restoration in the secondary dataset. **(A)** shows the three-way decision map; shaded regions indicate positive, boundary, and negative decision regions, and ellipses summarize treatment-level dispersion. **(B)** shows representative source images from the positive, boundary, and negative regions, with image ID, treatment status, mean PA/NA scores, response counts, and affective-restoration membership. Adapted from the open dataset by Navarrete-Hernandez and Laffan (2022), licensed under CC BY 4.0.

Figure 4 presents the empirical decision map and adds representative source images from the positive, boundary, and negative regions to make the three-way classification visually interpretable.

### 4.5 Results

The secondary analysis classified 99 urban-site image conditions into three regions, Equation 27 reports the resulting three-way region counts:


$$\begin{array}{l} |POS\left(C\right)|=3,\;|BND\left(C\right)|=87,\;|NEG\left(C\right)|=9 \end{array}$$ (27)

Only a small number of image conditions showed sufficiently high positive affect and sufficiently low negative affect to be accepted as affectively restorative. A small number fell into the negative region. Most images were classified into the boundary region, indicating mixed or moderate affective restoration rather than clear success or failure.

The positive region contained three image conditions, all associated with high positive affect and low negative affect. The three positive cases belonged to the G8 high-income site-image category, with successful-restoration memberships of 0.699, 0.683, and 0.679. These images can be interpreted as affectively successful exposure conditions under the selected loss function and thresholds.

The boundary region contained 87 image conditions. The highest boundary cases were close to the positive threshold but did not fully satisfy the acceptance criterion. These cases are especially important for design. They show relatively strong positive affect but remain below the acceptance threshold. In 3W-AREM, such images should not be rejected. Instead, they should be treated as candidates for adaptive regulation. Possible design actions may include improving visibility, adjusting vegetation arrangement, reducing perceived disorder, increasing spatial legibility, or modifying sensory intensity.

The negative region contained nine image conditions. These conditions showed low successful-restoration membership, mainly because positive affect was low and/or negative affect was high. They should not be accepted as affectively restorative under the present model. If used as design references, they would require substantial redesign rather than minor adjustment.

### 4.6 Treatment and site-category comparisons

To examine whether the three-way classification captures meaningful differences in the original dataset, image conditions were summarized by treatment marker. Control/placebo images and green-intervention images showed different affective-restoration profiles.

Green-intervention images showed higher mean positive affect, lower mean negative affect, and higher successful-restoration membership than control/placebo images. The control/placebo images had mean *PA* = 4.450, mean *NA* = 4.689, and mean *Pr*(*C*) = 0.383, with 0 positive, 11 boundary, and 7 negative images. The green-intervention images had mean *PA* = 5.534, mean *NA* = 3.697, and mean *Pr*(*C*) = 0.504, with three positive, 76 boundary, and two negative images. The difference in successful-restoration membership was statistically supported by complementary procedures: Welch test, Mann–Whitney *U*-test, bootstrap confidence interval, and permutation test. Green-intervention images showed a higher mean *Pr*(*C*) than control/placebo images, with a mean difference of 0.120, 95% bootstrap CI [0.072, 0.165], Welch *p* < 0.001, permutation *p* < 0.001, and Hedges *g* = 1.256. The three-way region distribution also differed by treatment, χ^2^(2) = 23.948, *p* < 0.001, Cramer's *V* = 0.492. A Fisher exact test on negative-region status showed that control/placebo images contributed disproportionately to the negative region, odds ratio = 0.040, *p* < 0.001.

As a supplementary raw-response-level robustness check, a mixed-effects model was fitted to normalized individual affect ratings with fixed effects for treatment, affect valence, and their interaction, random participant intercepts, and variance components for image condition and item label. This model was not used to replace the image-level design classification, but it addressed the non-independence of repeated ratings more directly. The green-intervention effect was negative for negative-affect items (*b* = −0.102, *p* < 0.001) and positive for positive-affect items after adding the treatment-by-valence interaction (*b* = 0.106). The interaction term was significant (*b* = 0.208, *p* < 0.001), supporting the descriptive pattern that green-intervention images were associated with lower negative-affect ratings and higher positive-affect ratings at the raw-response level.

**Figure 5:**
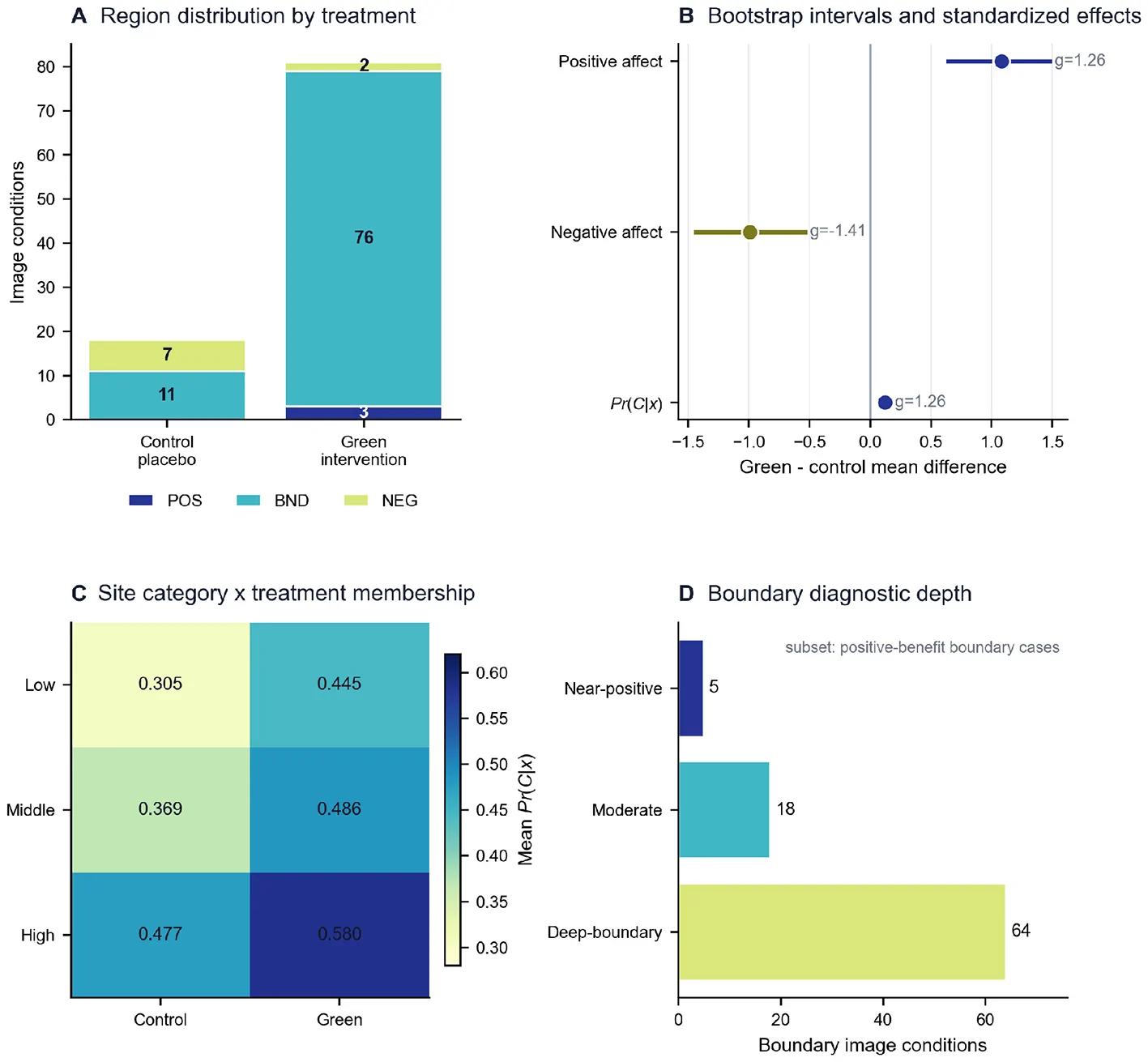
Empirical evidence dashboard for three-way restorative-environment classification. **(A)** The dashboard integrates region counts, **(B)** bootstrap effect intervals, **(C)** site-category membership, and **(D)** boundary diagnostic depth; precise numerical tests are reported in the accompanying tables.

Figure 5 integrates the main empirical summaries: region distribution by treatment, bootstrap intervals, site-category membership, and boundary diagnostic depth.

This result supports a nuanced interpretation. Small-scale green infrastructure appears to increase affective-restorative potential, but it does not automatically produce conditions that should be accepted as robustly restorative without further design evaluation. A green intervention may move an urban site away from the negative region, but many such interventions still require adaptive regulation before they can be treated as stable restorative design patterns.

Tables 10–12 report the descriptive statistics, inferential treatment comparisons, and three-way region counts underlying the visual summary in Figure 5.

**Table 10:** Descriptive statistics by treatment condition at the image-condition level.

Treatment	Images	PA mean	NA mean	*Pr*(*C*) mean	*Pr*(*C*) SD	Median *Pr*(*C*)
Control/placebo	18	4.450	4.689	0.383	0.093	0.371
Green intervention	81	5.534	3.697	0.504	0.096	0.503

**Table 11:** Inferential comparison between green-intervention and control/placebo image conditions.

Variable	Mean diff.	95% bootstrap CI	Welch *p*	Permutation *p*	Hedges *g*
PA	1.083	[0.644, 1.481]	< 0.001	< 0.001	1.256
NA	–0.993	[–1.436, –0.536]	< 0.001	< 0.001	–1.409
*Pr*(*C*)	0.120	[0.072, 0.165]	< 0.001	< 0.001	1.256

**Table 12:** Three-way region distribution by treatment condition.

Treatment	POS	BND	NEG
Control/placebo	0	11	7
Green intervention	3	76	2

The dataset also labels image/site categories as low-, middle-, or high-income. These labels should be interpreted as site or image-category descriptors rather than participant socioeconomic status, and they should not be used here to make individual-level social-fairness claims. The high-income site-image category showed the highest affective-restoration membership, while the low-income control/placebo category showed the lowest membership and the largest number of negative-region cases. Green interventions improved the low-income image category relative to its control/placebo category, but two low-income green-intervention images still fell into the negative region. This pattern suggests that site context remains important when interpreting affective responses to green infrastructure, but the present analysis cannot identify participant SES effects or neighborhood-level causal mechanisms.

The group comparison results should therefore be interpreted as evidence of affective improvement at the image level, not as proof that the images satisfy the full restorative-environment construct. The main empirical finding is not simply that green-intervention images performed better than controls. It is that improved affective membership still left most images in the boundary region. This is precisely the condition for which 3W-AREM is useful: a design intervention may reduce affective cost and increase affective benefit, but still require additional evidence on ART qualities, safety, attention, physiology, and embodied use before being accepted as a complete restorative design pattern.

### 4.7 Threshold sensitivity

Because three-way decision theory is explicitly cost-sensitive, classification should be examined under different loss settings. A sensitivity analysis was conducted using four loss scenarios: lenient, main, strict, and very strict. Under the lenient scenario, 32 images entered the positive region, 43 entered the boundary region, and 24 entered the negative region. Under the main scenario, three entered the positive region, 87 entered the boundary region, and nine entered the negative region. Under strict and very strict scenarios, all images entered the boundary region, reflecting the high evidentiary standard required before accepting affective restoration.

**Figure 6:**
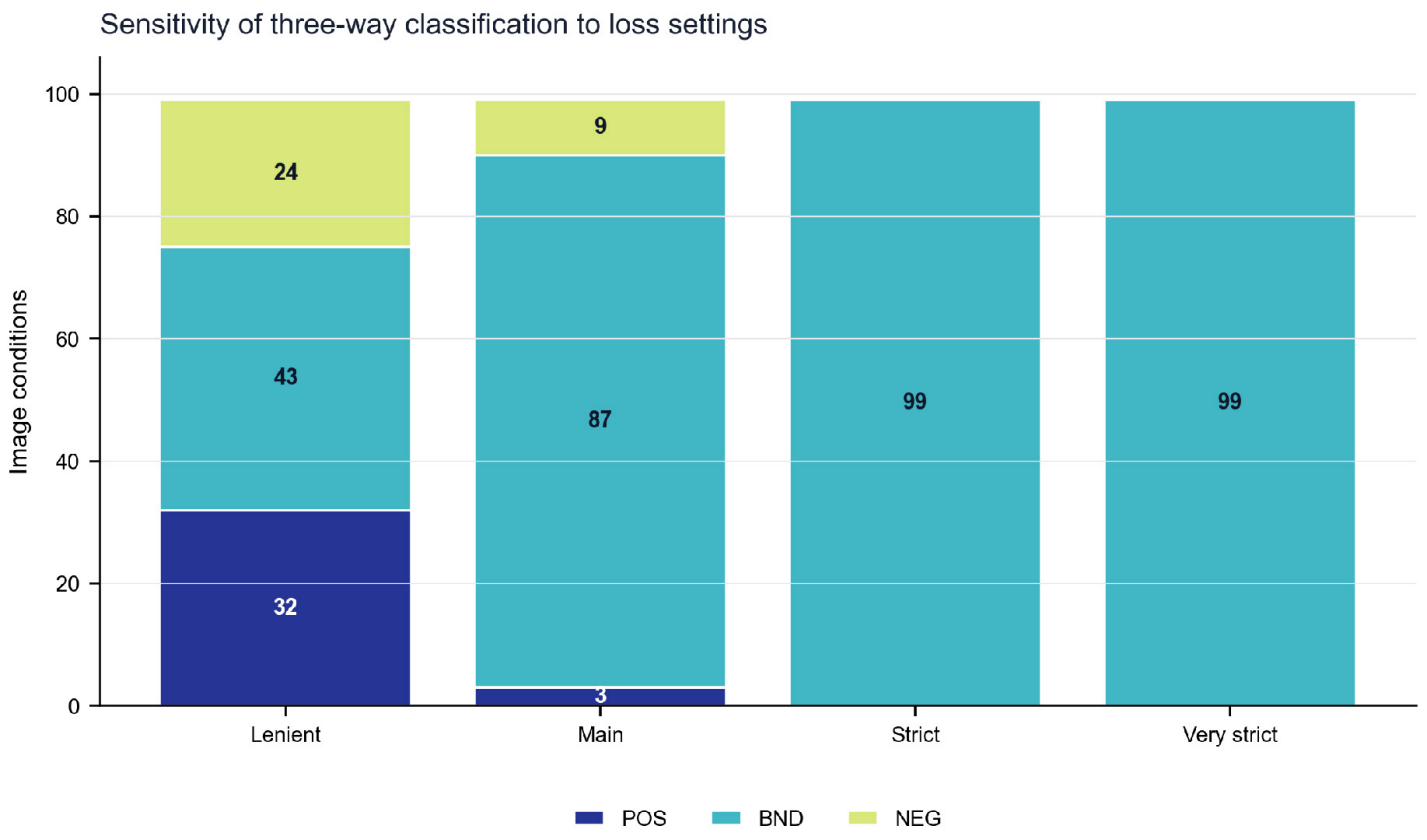
Sensitivity of three-way classification to alternative loss settings.

Table 13 reports the loss vectors, thresholds, and resulting region counts for the four sensitivity scenarios, while Figure 6 visualizes the same transition pattern.

**Table 13:** Loss-parameter sensitivity and resulting three-way classifications.

Scenario	Loss vector	α	β	POS	BND	NEG
Lenient	(0,5,8,0,2,8)	0.545	0.400	32	43	24
Main	(0,4,8,0,2,10)	0.667	0.333	3	87	9
Strict	(0,3,9,0,2,12)	0.769	0.250	0	99	0
Very strict	(0,2,10,0,2,14)	0.857	0.200	0	99	0

Loss parameters are ordered as (λ_*PP*_, λ_*BP*_, λ_*NP*_, λ_*NN*_, λ_*BN*_, λ_*PN*_).

**Figure 7:**
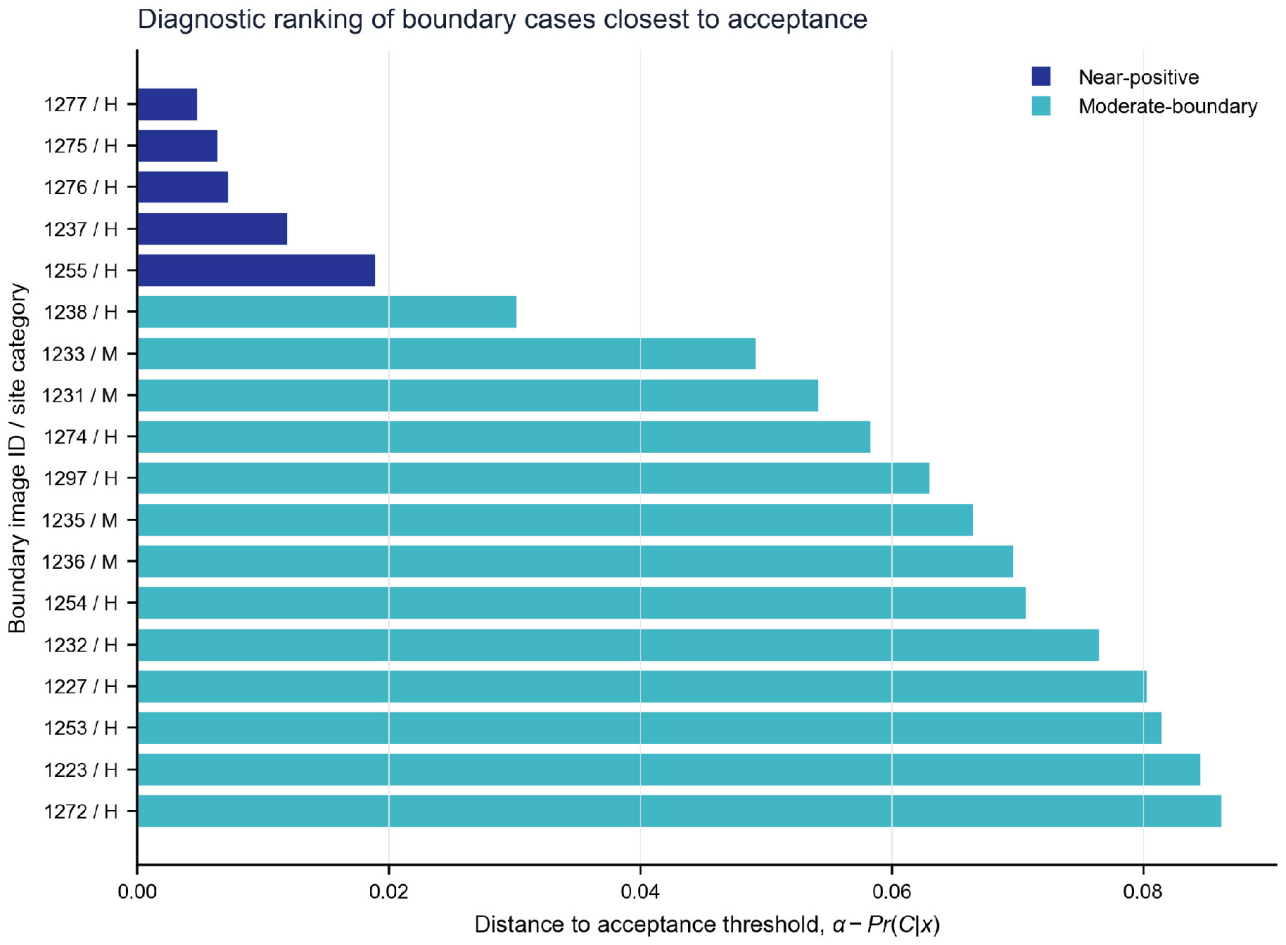
Diagnostic ranking of boundary cases closest to acceptance.

Figure 7 ranks boundary-region cases by distance to the acceptance threshold, showing which cases are closest to becoming acceptable after targeted benefit improvement.

This sensitivity is not a weakness of the model. It shows that 3W-AREM can be adjusted to different design contexts. A hospital, school, elder-care facility, or mental-health setting may require a stricter loss function than a low-stakes recreational environment. The model, therefore, allows design decisions to be calibrated according to the ethical and practical consequences of false acceptance, unnecessary regulation, and false rejection. Boundary-region diagnostics further showed that five cases were near-positive, 18 were moderate-boundary cases, and 64 were deep-boundary cases. In the current affective dataset, all boundary cases were benefit-limited rather than cost-limited, meaning that positive affect was the binding component in the fuzzy-min membership function. This suggests a concrete design interpretation: many boundary urban-site images already met the low-negative-affect constraint, but required stronger positive restorative benefit before they could be accepted as stable restorative exposure patterns.

The transition pattern across loss settings clarifies what changes when thresholds become stricter. From the lenient to the main scenario, 29 previously positive cases moved into the boundary region, 15 previously negative cases moved into the boundary region, and only three positive and nine negative cases retained their extreme classifications. From the main to the strict scenario, all three positive cases and all nine negative cases moved into the boundary region. Across the four settings, the most common pathway was stable boundary membership (43 cases), followed by positive-to-boundary movement (29 cases), negative-to-boundary movement (15 cases), stable early-negative then boundary movement (nine cases), and stable early-positive then boundary movement (three cases). Thus, stricter loss settings mainly reduce premature acceptance and rejection rather than reversing the core conclusion that many images are design-actionable boundary cases.

Table 14 summarizes how boundary-region patterns should be translated into design diagnosis rather than treated as statistical uncertainty only.

**Table 14:** Design interpretation of boundary-region diagnostic patterns.

Boundary pattern	Computational meaning	Design implication
Benefit-limited boundary	*B*(*x*) ≤ *LowNA*(*x*); positive affect is the binding component	Increase restorative affordance through richer vegetation, seating quality, material warmth, shade, visual interest, compatibility, or sense of invitation.
Cost-limited boundary	*LowNA*(*x*) < *B*(*x*); negative affect or discomfort is the binding component	Reduce affective cost through improved visibility, maintenance, safety, legibility, noise control, reduced disorder, or lower sensory intensity.
Near-positive boundary	α−*Pr*(*C*|*x*) is small	Prioritize low-cost targeted adjustment and re-evaluation because the exposure condition is close to acceptance.
Deep-boundary case	α−*Pr*(*C*|*x*) is large but *Pr*(*C*|*x*)≥β	Conduct more substantial redesign or collect additional environmental variables before acceptance.

## 5 Discussion

### 5.1 Main findings

This study proposed 3W-AREM as a decision-theoretic framework for classifying and regulating restorative environmental exposure under psychological uncertainty. The model was developed in response to a recurring problem in restorative environment research and biophilic design: natural or nature-like exposure is often beneficial, but its effects are not universal, linear, or free from contextual constraints. Instead, restorative response may vary across exposure duration, sensory composition, spatial configuration, perceived safety, baseline psychological state, and measurement channel.

The central theoretical move of 3W-AREM is to distinguish three decision regions. The positive region represents exposure conditions that can be accepted as restorative. The negative region represents exposure conditions that should be rejected or structurally redesigned. The boundary region represents mixed or uncertain restorative response. This boundary region is the main contribution of the model. It allows environments with partial restorative potential to be treated as design-actionable rather than as failed or noisy cases. In practical terms, a boundary decision means that the designer should diagnose the limiting component, adjust the relevant design granule, and retest the exposure condition rather than prematurely declaring restoration failure.

The secondary data demonstration illustrated the feasibility of this three-way perspective. Using an open dataset on small-scale green infrastructure and affective well-being in urban sites, 99 image conditions were classified according to fuzzy membership in a successful affective-restoration class. Only three image conditions fell into the positive region, nine fell into the negative region, and 87 fell into the boundary region. This result suggests that many urban-site image conditions are affectively intermediate. They may generate some degree of positive affect or reduced negative affect, but not strongly enough to be accepted as robustly affective-restorative under a cost-sensitive decision rule.

This pattern is important because it reflects the practical reality of environmental design. Most urban green interventions are neither clearly successful nor clearly ineffective. They often sit between promise and limitation: they may be greener but still visually disordered, emotionally positive but not fully calming, interesting but not fully comfortable, or affectively improved but not yet strong enough to justify acceptance as restorative design patterns. Three-way decision theory provides a formal way to preserve this intermediate status and convert it into a design problem.

### 5.2 Theoretical contribution

The first contribution of this study is to reframe restorative environmental exposure as a decision problem under uncertainty. Much existing research evaluates environments through mean differences, correlations, or binary comparisons between natural and urban conditions. These approaches are valuable for identifying general effects, but they provide limited guidance when an environment produces mixed outcomes. A three-way model is useful because it separates the question of evidence from the question of action. An exposure condition does not need to be fully accepted or rejected when evidence is uncertain; it can be placed in a boundary region for further regulation.

This approach is consistent with recent evidence showing heterogeneity in restorative environment effects. Meta-analytic work on nature exposure, attention restoration, physiological stress recovery, and nature-based interventions suggests that environmental benefits depend on exposure duration, medium, user population, and outcome type. Urban design studies further show that naturalness interacts with perceived safety, maintenance, visibility, sensory conditions, and social affordances. These findings imply that restorative exposure is not a fixed property of natural elements. Rather, it is a situated user-environment response.

3W-AREM contributes to this literature by formalizing the mixed-response category. Instead of treating heterogeneity as a methodological inconvenience, the model treats it as theoretically meaningful. The boundary region marks the conditions under which restorative potential exists but remains incomplete, unstable, or constrained by cost.

### 5.3 Contribution to cognitive aesthetics

The model also contributes to cognitive aesthetics by emphasizing restorative exposure as a valuation process. Environmental experience is shaped by sensory input, affective appraisal, bodily state, learned expectation, and behavioral context. A green space may be pleasant because it affords calm, safe, and meaningful sensory valuation; it may be adverse if the same sensory features are interpreted as unsafe, excessive, or incompatible with the user's current state.

From this perspective, restorativeness is not reducible to objective green quantity or visual naturalness. It depends on how the environment is valued by the perceiver. The fuzzy definition of successful affective restoration reflects this idea by requiring both positive affective benefit and low affective cost. High positive stimulation alone is not sufficient. A public space that is exciting but also frightening, confusing, or overwhelming should not be accepted as restorative without further qualification.

### 5.4 Methodological contribution

Methodologically, this study introduces a cost-sensitive classification structure into restorative environmental design. Traditional binary classification may force researchers to choose between acceptance and rejection even when evidence is mixed. In contrast, three-way decision theory introduces an explicit deferred or adaptive action. This is useful because environmental design errors have asymmetric costs.

Accepting an environment as restorative when it produces discomfort, unsafety, or negative affect may mislead design practice and expose users to unsuitable conditions. Rejecting an environment that has partial restorative potential may also be costly because it overlooks opportunities for low-cost improvement. The boundary decision balances these risks by allowing further evaluation or adaptive regulation.

The model also links three-way decision theory with granular computing. This is important because classification alone is insufficient for design. Once an exposure condition is placed in the boundary region, the designer must know what can be adjusted. The proposed granularity hierarchy decomposes restorative exposure into medium, environmental type, spatial configuration, sensory composition, parameter intensity, and user-state adaptation. This structure makes it possible to move from classification to intervention.

### 5.5 Design implications

The practical implication of 3W-AREM is that restorative design should be understood as adaptive regulation rather than simple addition of natural elements. Designers should not assume that increasing vegetation, extending exposure, adding water, or intensifying natural stimuli will automatically improve psychological restoration. Instead, they should evaluate whether the intervention increases restorative benefit while keeping affective, cognitive, and safety costs within acceptable limits.

For urban green infrastructure, this means that boundary-region spaces may require targeted adjustments rather than complete rejection. If positive affect is moderate but negative affect remains high, designers may need to improve maintenance, reduce visual disorder, increase visibility, or address perceived safety. If positive affect is low but negative affect is also low, the space may be safe but insufficiently engaging, requiring richer vegetation, better seating, more natural materials, or stronger compatibility with user needs. If both positive and negative affect are high, the space may be stimulating but unstable, requiring regulation of sensory intensity or spatial complexity.

The real-data demonstration illustrates this design logic at the image-condition level. Most image conditions fell into the boundary region, suggesting that many urban sites may have partial affective potential but require more precise design diagnosis. Future applications should combine affective data with environmental measurements such as vegetation density, visibility, disorder, soundscape, enclosure, and exposure duration. This would allow each boundary condition to be linked to specific restorative exposure granules.

Three design scenarios illustrate how this logic can be used (Table 15). These scenarios are not intended as universal prescriptions. They show how 3W-AREM converts a boundary-region result into a concrete diagnosis and a re-testable design scheme for settings that are common in restorative-environment research and practice (Yin et al., 2018; Hasebe and Harada, 2025; Liu and Lee, 2026; Sedghikhanshir and Montelli, 2026).

**Table 15:** Targeted design schemes for typical boundary-region restorative environments.

Scenario	Typical boundary pattern	Key additional indicators	Granular diagnosis	Targeted optimization and re-testing
Hospital courtyard or lobby	Calmness improves, but privacy, perceived safety, orientation, or patient comfort remains insufficient	PRS/RPRS, perceived safety, privacy, HRV/EDA, dwell time, and staff/patient usability	GL3 spatial configuration and GL5 parameter intensity: refuge, sightline, seating distance, shade, circulation, and planting height	Increase controllable refuge without visual obstruction; improve wayfinding, seating choice, shade, and maintenance cues; re-test affect, perceived restorativeness, safety, and physiological regulation before acceptance
Urban pocket park	Negative affect is low, but positive affect, fascination, coherence, or invitation is not high enough for acceptance	Positive/negative affect, fascination, coherence, maintenance, soundscape, visibility, and behavioral mapping	GL4 sensory composition and GL5 parameter intensity: vegetation layering, material warmth, acoustic comfort, visual complexity, and seating affordance	Add legible paths, layered planting, comfortable seating, maintenance cues, and moderate sensory richness; re-test membership and classify whether the site crosses α without increasing cost
Indoor restorative space	Natural elements are present, but attention recovery, comfort, or compatibility with work/rest tasks remains incomplete	PRS/RPRS, attention task, workload, lighting comfort, thermal/acoustic comfort, eye tracking, and HRV	GL2 environmental type, GL4 sensory composition, and GL6 user-state adaptation: daylight, view, plant density, material texture, break duration, and user need	Calibrate daylight, vegetation density, natural materials, acoustic shielding, and exposure duration; compare baseline and optimized conditions using affect, attention, comfort, and physiological indicators

**Figure 8:**
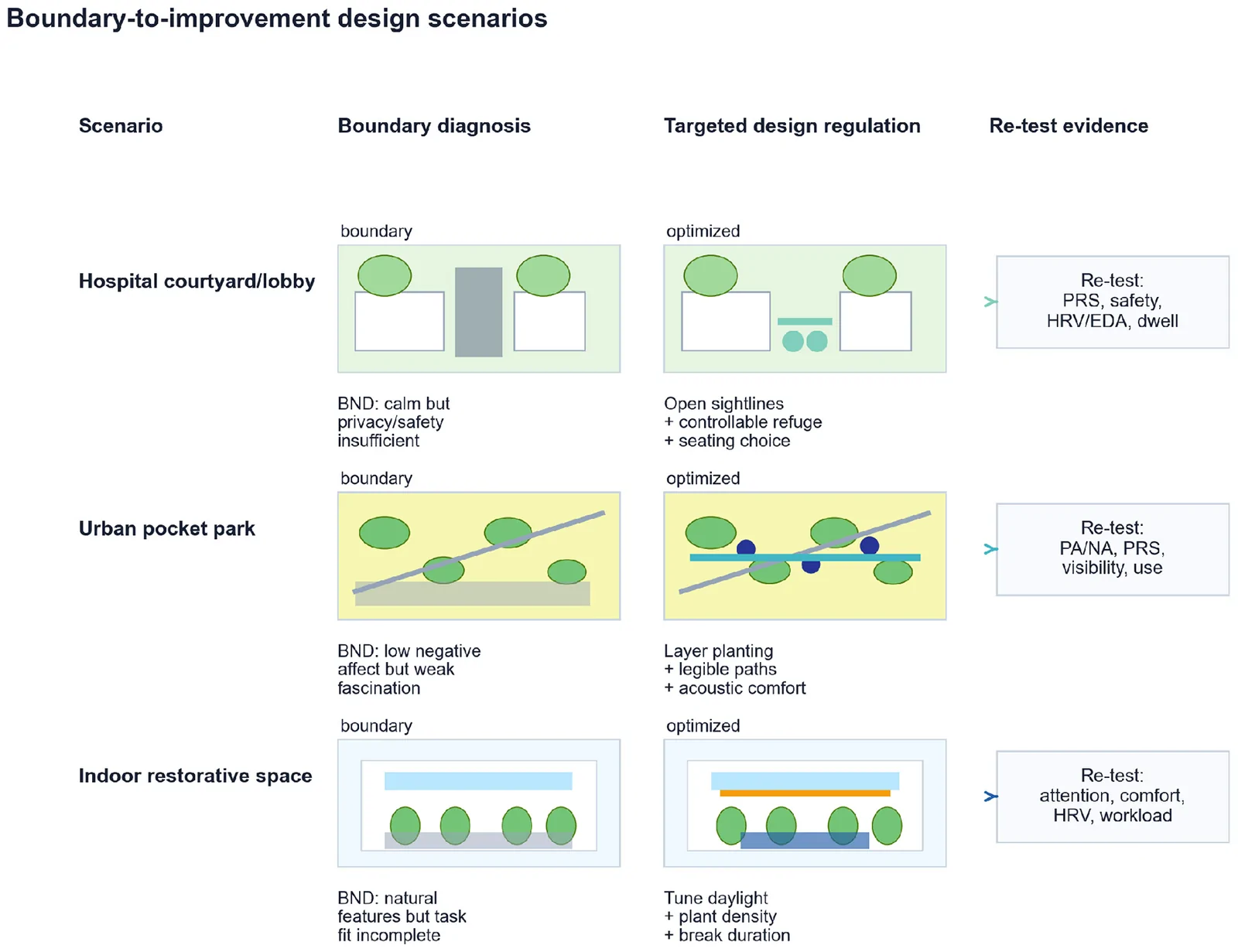
Boundary-to-improvement design scenarios. The three rows illustrate how 3W-AREM can convert a boundary-region classification into diagnosis, targeted design optimization, and re-testing for a hospital courtyard or lobby, an urban pocket park, and an indoor restorative space. The diagrams are schematic design vignettes derived from the model and the scenario logic in Table 15; they are not additional empirical results.

Figure 8 extends Table 15 by showing how a boundary diagnosis can be translated into targeted design regulation and re-testing evidence in three typical scenarios. The figure is schematic rather than a new empirical test; its purpose is to make the central “Boundary → Design Improvement” logic visually explicit.

A data-to-design brief can therefore be written in a directly actionable format: “The image condition is in the boundary region because μ_*C*_(*x*) is benefit-limited; low negative affect is adequate, but positive affect remains below α. Increase inviting restorative affordances at GL4/GL5, retest PA/NA and perceived restorativeness, and accept only if the adjusted condition crosses α without increasing affective cost.” This is the practical value of treating boundary membership as a design instruction rather than a statistical inconvenience.

### 5.6 Limitations and future research

The limitations of this study should be read in relation to its article type. The paper proposes a decision-theoretic framework and provides a secondary image-level affective demonstration. It does not claim to provide a complete empirical validation of restorative environments.

Table 16 separates current limitations and future research into short-term and long-term items so that readers can distinguish improvements that are feasible in near-term secondary or replication studies from longer-term embodied, neurophysiological, and intervention studies.

**Table 16:** Limitations and future research agenda.

Time horizon	Current limitation	Recommended future work
Short-term	The open dataset contains image-based affective responses but not direct PRS/RPRS scores, ART qualities, attention tasks, physiological measures, safety ratings, soundscape data, movement, or exposure duration	Replicate the analysis with datasets that include perceived restorativeness, being away, fascination, coherence, compatibility, attention recovery, safety, and comfort indicators; report the affective layer separately from complete restoration
Short-term	The secondary demonstration uses image-condition aggregation because each participant generally rated one PANAS-like adjective rather than a complete affect profile for every image	Add confirmatory mixed-effects or hierarchical models when raw data structure permits; include participant, image, item, and site random effects; report both image-level design classifications and individual-level uncertainty
Short-term	Thresholds and losses are theoretically motivated and transparent, but not yet calibrated for a specific design institution or user population	Use expert elicitation, Delphi panels, Bayesian decision analysis, or stakeholder risk analysis to calibrate α, β, and the loss matrix for hospitals, workplaces, schools, parks, or digital exposure settings
Long-term	Image exposure has lower ecological validity than embodied field, VR, AR, or real-time building exposure	Test the evaluation-diagnosis-optimization-retesting loop in field studies, immersive VR, and living-lab environments, with repeated exposure and behavioral outcomes
Long-term	The neuroaesthetic extension is conceptual and not estimated in the present paper	Pre-register neural hypotheses, ROIs, graph metrics, and exclusion rules; use EEG, fMRI, fNIRS, eye tracking, and physiological monitoring only as complementary evidence; control high-dimensional features with regularization and independent-sample validation
Long-term	The model has not yet been tested as an adaptive design intervention	Conduct experimental boundary-region optimization studies to verify whether targeted changes in GL1–GL6 move exposure units from *BND*(*C*) to *POS*(*C*) without increasing affective, cognitive, physiological, or safety-related costs

These limitations also define the model's applicable scope. The current empirical results apply to image-level affective restoration in urban-site stimuli, not to full restorative recovery in embodied environments. Full 3W-AREM application requires multimodal evidence, including ART qualities and design constraints. The central claim supported here is therefore methodological: three-way decision theory can formalize mixed restorative evidence and translate boundary-region cases into adaptive design action.

### 5.7 Future neuroaesthetic and brain-connectivity extension

The framework also offers a future-oriented bridge to neuroaesthetic and brain-connectivity research, but this bridge should be read as a hypothesis-generating extension rather than as a finding of the present secondary analysis. Boundary-region environments could be tested as cases in which sensory engagement and valuation are not fully aligned. For example, visual and attention systems may respond strongly to environmental complexity while threat-appraisal or cognitive-control systems signal discomfort or overload. Conversely, low-arousal environments may reduce threat signals but fail to support positive valuation. These claims remain hypotheses because the present study does not analyze EEG, fMRI, fNIRS, eye-tracking, HRV/EDA, or physiological connectivity data.

For future neuroaesthetic work, a restorative exposure condition can be represented as a network valuation process, but only when independent neural or physiological data are actually collected. Let the optional neuroaesthetic response network be:


$$\begin{array}{l} N=\left(V,E,W\right) \end{array}$$ (28)

where *V* denotes pre-registered nodes or regions of interest, *E* denotes functional or effective connections, and *W* = [*w*_*ij*_] denotes the weighted connectivity matrix. Candidate systems may include visual processing, attention, executive control, threat appraisal, valuation, and memory or meaning networks (Chatterjee and Vartanian, 2014; Coburn et al., 2017; Pearce et al., 2016).

For a given exposure unit (*x*), Equations 28–30 define the optional future neural response network, connectivity-informed membership model, and boundary ambiguity index:


$$\begin{array}{l} Pr_{N}\left(C|x\right)=\sigma \left(\omega_{0}+\overset{m}{\underset{k=1}{\sum }}\omega_{k}z_{k}\left(x\right)+\underset{i<j}{\sum }\eta_{ij}w_{ij}\left(x\right)\right) \end{array}$$ (29)

where *z*_*k*_(*x*) are psychological, physiological, or design features, *w*_*ij*_(*x*) are pre-specified connectivity features, ω_*k*_ and η_*ij*_ are model weights, and σ(·) is a logistic transformation. This equation is not estimated in the present study. It is included only to show how future 3W-AREM studies could integrate neuroaesthetic data without replacing psychological evidence. Because connectivity features are high-dimensional and often collinear, future implementation must standardize features, pre-register hypotheses, regions, and graph metrics, use regularized or hierarchical models, correct for multiple testing when appropriate, and validate performance on independent or held-out exposure conditions. A future boundary ambiguity index can then be defined as:


$$\begin{array}{l} NAI\left(x\right)=1-|2Pr_{N}\left(C|x\right)-1| \end{array}$$ (30)

where higher *NAI*(*x*) indicates greater ambiguity around the future neural membership estimate. Different neuroimaging methods would test different parts of this model. EEG studies are better suited to temporal dynamics, attentional engagement, affective arousal, frequency-specific connectivity, and repeated design-state monitoring; fMRI is better suited to spatially resolved network localization. In both cases, neural evidence should complement, rather than replace, self-report, behavioral, physiological, and design-variable measurements.

**Figure 9:**
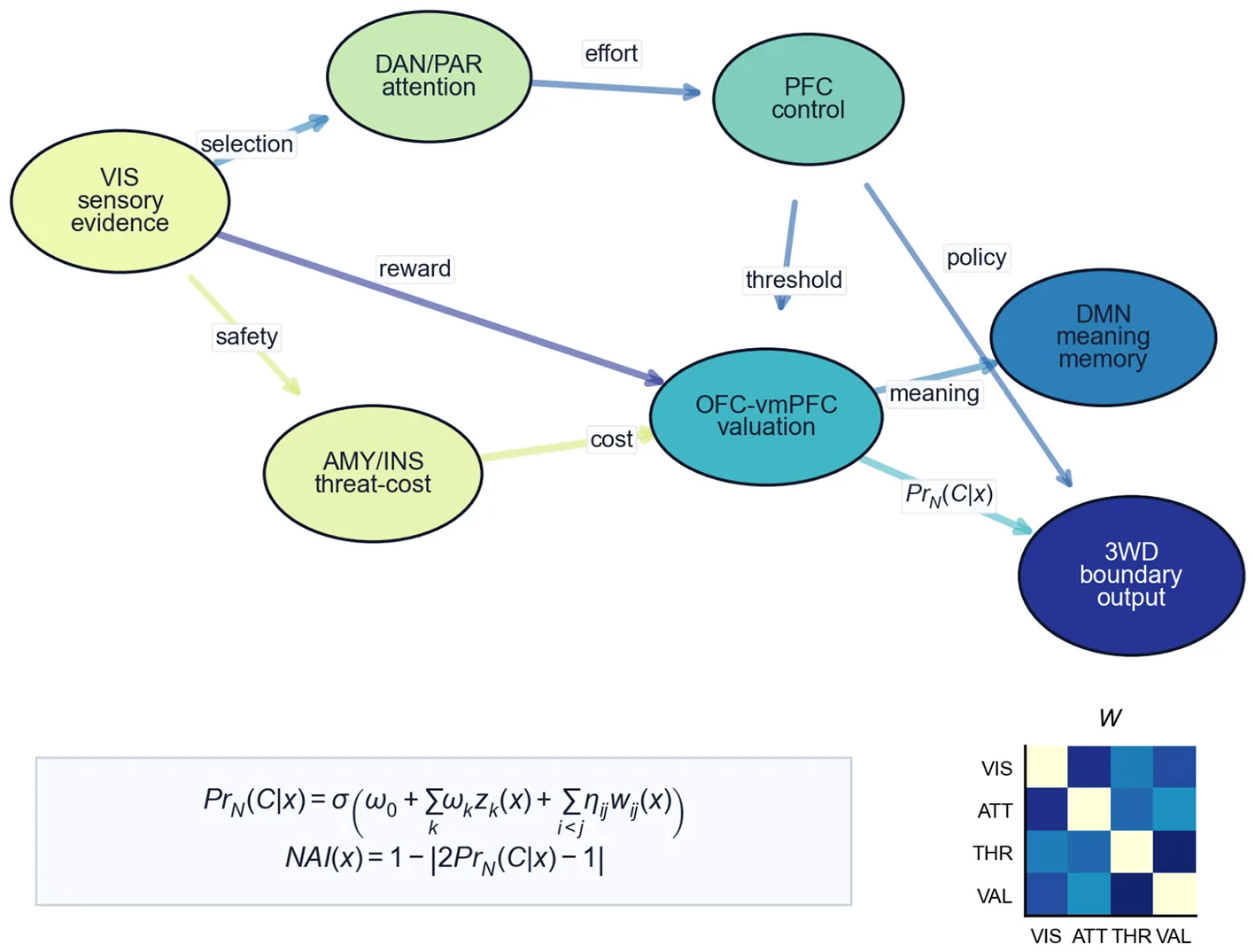
Hypothesis-generating neuroaesthetic and brain-connectivity extension of 3W-AREM. The figure shows how future studies could connect exposure granules, psychological and physiological indicators, pre-registered neural features, and independent validation. It is included as a future research model only; no neural data are analyzed in the present secondary demonstration.

Figure 9 illustrates this future extension as a pre-registered, validation-oriented pathway rather than as evidence already obtained in the present study.

## 6 Conclusion

This study proposed the Three-Way Adaptive Restorative Exposure Model as a framework for understanding and regulating restorative environmental exposure under psychological uncertainty. The model argues that restorative environments should not be evaluated only as beneficial or ineffective. Many environments produce mixed affective, cognitive, or physiological responses and therefore require a third design status.

By integrating three-way decision theory with granular computing, 3W-AREM classifies exposure conditions into positive, boundary, and negative regions. The positive region supports acceptance, the negative region supports rejection or structural redesign, and the boundary region supports adaptive regulation. This boundary region is the main design contribution of the model because it identifies environments with partial restorative potential.

A secondary image-level affective demonstration using an open urban green-infrastructure dataset illustrated the feasibility of this approach. Most image conditions fell into the boundary region, suggesting that affective responses to urban green-intervention images are often intermediate rather than clearly successful or clearly adverse. Green-intervention images showed improved affective-restoration membership compared with control/placebo images, but most still required boundary-region interpretation. This finding supports the need for models that can formally represent mixed environmental responses without equating affective improvement with complete attentional, physiological, or embodied restoration.

Operationally, the model suggests a six-step design workflow: measure benefit and cost indicators; compute membership in the target restorative class; classify the exposure condition into positive, boundary, or negative regions; locate the relevant design granule; adjust the boundary condition through targeted design action; and retest the adjusted exposure before acceptance. Overall, the proposed framework contributes to environmental psychology, cognitive aesthetics, and evidence-based design by offering a cost-sensitive and design-actionable method for translating restorative environment evidence into adaptive intervention decisions. The framework also offers a future-oriented bridge to neuroaesthetic and brain-connectivity research, but the present paper does not provide neural empirical evidence. Future studies should extend the model using VR, field experiments, physiological measures, personalized decision thresholds, pre-registered neural hypotheses, and independent-sample brain-connectivity validation. In this way, restorative environmental design can move beyond the question of whether nature is generally beneficial and toward a more precise question: which exposure, for which user, under which conditions, and at what level of adaptive regulation?
